# A viral noncoding RNA is a master regulator of gene expression that defines host cell identity and function

**DOI:** 10.1093/nar/gkag472

**Published:** 2026-05-11

**Authors:** Carlos Gorbea, Donald Yuhui Sim, Xavier Roca, Demián Cazalla

**Affiliations:** Department of Biochemistry, University of Utah, Salt Lake City, UT 84112, United States; School of Biological Sciences, Nanyang Technological University, 637551, Singapore; School of Biological Sciences, Nanyang Technological University, 637551, Singapore; Department of Biochemistry, University of Utah, Salt Lake City, UT 84112, United States

## Abstract

Viruses extensively reprogram the host cell to promote infection and persistence, yet the contribution of noncoding RNAs (ncRNAs) to this viral reengineering remains largely unknown. Here, we show that HSUR1, a viral Sm-class ncRNA abundantly expressed by the oncogenic *Herpesvirus saimiri*, functions as a master regulator of host gene expression. HSUR1 interacts with hundreds of cellular messenger RNAs (mRNAs) that encode factors involved in RNA metabolism, immune cell differentiation, and apoptosis, thereby rewiring the host transcriptome to control both cell survival and fate. Mechanistically, we show that HSUR1 acts as an RNA adaptor that coordinates the recruitment of the host microRNA miR-142-3p and AU-rich element–binding proteins to target mRNA 3′ untranslated regions, redirecting post-transcriptional regulation. These findings reveal that a single viral ncRNA can orchestrate global remodeling of host gene expression, uncovering an RNA-based strategy for viral control of cell identity and function.

## Graphical Abstract

**Graphical Abstract:**
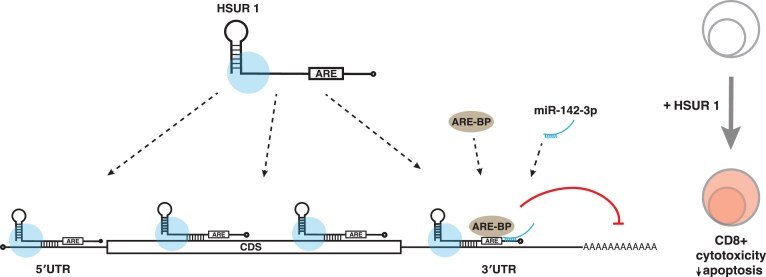

## Introduction

Successful viruses must do more than enter cells—they must reengineer them. To persist within their hosts, viruses reshape gene expression, signaling, and immune recognition, creating a cellular environment that favors their replication or long-term survival. This reprogramming is often mediated by viral proteins that co-opt key host pathways. However, many viruses also produce abundant noncoding RNAs (ncRNAs) whose contribution to the large-scale reorganization of host regulatory networks remains largely unknown.

*Herpesvirus saimiri* (HVS), a γ-herpesvirus that establishes latency in T cells of the squirrel monkey (*Saimiri sciureus*) and causes aggressive leukemias and lymphomas in other New World primates such as the common marmoset (*Callithrix jacchus*) [1], expresses seven small nuclear, U-rich noncoding RNAs (HSURs) [2–5], which are among the most abundant viral transcripts in infected T cells. Although these Sm-class RNAs have been known for decades, their biological roles have remained largely enigmatic.

Functions have so far only been assigned to HSUR1 and HSUR2. Both ncRNAs directly associate with host microRNAs (miRNAs): HSUR1 binds miR-142-3p and miR-27, while HSUR2 interacts with miR-142-3p and miR-16 [6]. HSUR2 functions as a miRNA adaptor, recruiting bound miRNAs to 3′ untranslated regions (3′UTRs) of cellular messenger RNAs (mRNAs) to repress their expression and modulate host pathways such as apoptosis [7, 8]. In contrast, HSUR1 engages miR-27 to trigger its degradation via target-directed microRNA decay (TDMD) [6], a process that involves a conformational change in the miRNA-associated argonaute protein rendering it susceptible to ubiquitin-mediated proteolysis [9–11]. This activity depends on prior binding of miR-142-3p to the 5′ end of HSUR1, which allosterically activates HSUR1’s ability to induce TDMD [12].

**Figure 1.**
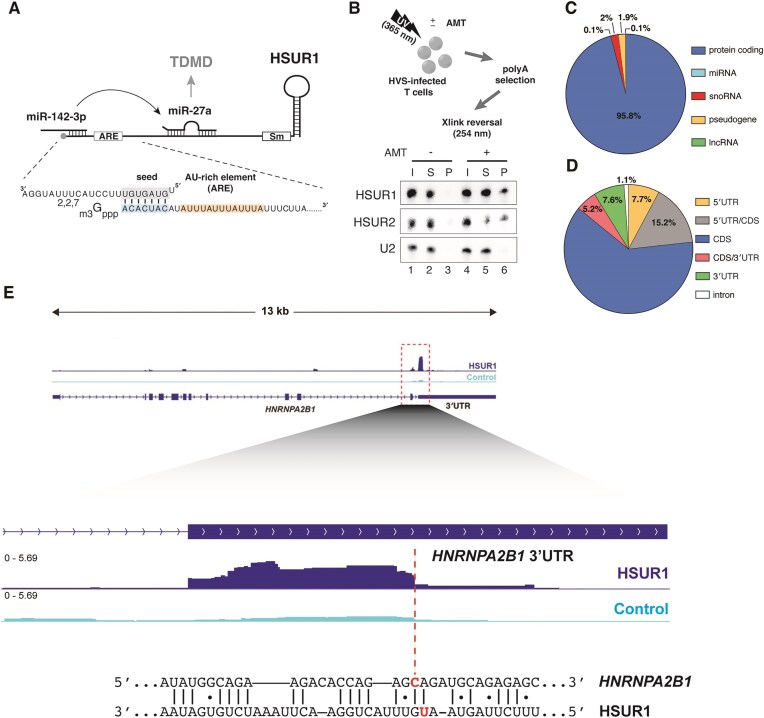
HSUR1 binds host mRNAs in infected cells. (**A**) Schematic diagram of HSUR1 and interacting miRNAs. Sequences corresponding to HSUR1’s ARE (light orange box) and miR-142-3p binding (light blue box) site, and miR-142-3p partial sequence (seed sequence in light gray box) are shown in detail. Sm: Sm-binding site. TDMD: target-directed miRNA decay. (**B**) Northern blot analyses of polyA+ RNA from HVS-transformed marmoset T cells (cj319-WT) that were *in vivo* crosslinked in the absence (lanes 1–3) or presence (lanes 4–6) of psoralen (AMT). I: input (1%); S: supernatant (1%); P: pellet (100%). (**C**) RNA transcript types interacting with HSUR1 revealed by iRICC. (**D**) Location of peaks corresponding to binding sites in HSUR1 target mRNAs. (**E**) Representative iRICC track of *HNRNPA2B1* mRNA for HSUR1 (blue) and Control (cyan) samples. Red dashed box indicates peaks of aligned sequencing reads present exclusively in 3′UTRs. HSUR1 and Control tracks are shown at the same scale. Numbers in zoomed-in tracks indicate relative abundance normalized by unique alignment read count. Dashed red line in zoomed-in view denotes abrupt drop of aligned reads indicating site of crosslinking. Predicted base pairing between HSUR1 and target sequences adjacent to the site of crosslinking are shown. Putative psoralen-crosslinked nucleotides are shown in red.

In addition to its miRNA-binding sites, HSUR1 contains an AU-rich element (ARE) near its 5′ end (Fig. 1A), a cis-regulatory motif that mediates RNA turnover and translation control of mRNAs [13]. HSUR1 binds several ARE-binding proteins (ARE-BPs) such as heterogenous ribonucleoprotein (hnRNP) D, Human antigen R (HuR), and tristetraprolin (TTP) [14, 15]. The HSUR1 ARE contributes to the RNA’s own stability, indicating that it functions as a bona fide regulatory element [16]. Yet, HSUR1 and HSUR2 do not alter the abundance of host ARE-containing mRNAs [14], suggesting that their AREs do not simply titrate host ARE-BPs. These observations raise the possibility that the combination of Sm-class architecture, miRNA interaction, and ARE motif endows HSUR1 with additional, unexplored functions in host–virus interactions.

We set out to understand the role of HSUR1 in HVS latency. We found that HSUR1 directly engages host mRNAs through RNA–RNA base pairing. Using individual-nucleotide resolution RNA–RNA interaction mapping (iRICC) [8], we identified hundreds of cellular mRNAs that interact with HSUR1 and defined the sequences mediating these contacts. We show that HSUR1 recruits miR-142-3p and ARE-BPs to target 3′UTRs, forming a tripartite complex that destabilizes transcripts through a mechanism utilizing both miR-142-3p and the HSUR1 ARE motif. Through this mechanism, HSUR1 acts as an RNA scaffold that links two central post-transcriptional regulatory systems—miRNAs and ARE-BPs—to coordinately rewire host gene expression. A large fraction of HSUR1 target mRNAs encodes regulators of RNA metabolism, chromatin organization, and transcription, suggesting that HSUR1 functions as a master coordinator of gene expression. Consequently, HSUR1 profoundly reshapes the transcriptome of HVS-transformed T cells, altering the expression of key transcription factors that control T-cell fate and identity. These findings reveal that a single viral ncRNA can orchestrate global remodeling of host gene expression, uncovering an RNA-based strategy by which viruses reengineer the host cell.

## Materials and methods

### Plasmids and HVS bacmids

Plasmids for stable expression of HSUR1, firefly, and *Renilla* luciferase reporters have been already described [8, 12]. Fragments corresponding to partial ADAR, HNRNPA2B1, FOXN3, PIP4K2B, PPFIA1, UBA2, ILRUN, and JARID2 3′UTRs were amplified by polymerase chain reaction (PCR) from marmoset genomic DNA prepared from cj319-WT cells and cloned between the *luc2* gene and the bGH polyA in pLenti-TK-Firefly-Control [8]. The plasmids pBS-GFP-U1 and pBS-GFP-U1-HSUR1 for transient expression of HSUR1 and green fluorescent protein (GFP) were obtained by PCR amplification of a fragment containing either the U1 snRNA promoter or the U1 promoter, HSUR1 sequence, and the U1 3′-end box using pUC-U1-HSUR1 [16] as template and cloned into the *Bam*HI and *Eco*RI sites in pBS-GFP [7]. Mutant versions of the plasmids described earlier were obtained by overlap extension PCR and confirmed by sequencing. To generate a plasmid for transient expression of ZFP36L2 and selection of transfected cells based on mCherry2 expression, a fragment containing the human CMV enhancer promoter, mCherry2, and the cleavage and polyadenylation signal of the simian virus 40 (SV40) was generated by digestion of the plasmid mCherry2-N1 (Addgene #54517) with *Nde*I and *Psi*I and inserted into the *Nde*I and *Psi*I sites of the plasmid ZFP36L2 (Addgene #156149) to create the plasmid ZFP36L2-mCherry2. ZFP36L2-mCherry2 was digested with *Pme*I and *Spe*I to release a complementary DNA (cDNA) fragment encoding ZFP36L2 fused to a V5-epitope, blunted with Klenow polymerase, and re-ligated to generate the plasmid Control-mCherry2.

The bacmid construct HVS-BAC-GFP-WT expressing wild-type (WT) HVS strain A11 (NCBI:txid10383; PRJNA146221; GEO:GSE31065; RRID:NCBITaxon_10383) was previously generated by *en passant* mutagenesis [7]. To generate a bacmid for expression of HVS A11 lacking HSUR1, we first generated a plasmid that contains the first ∼ 7 kilobases (kb) of HVS A11 genome by insertion of a HVS A11 genomic fragment generated by overlapping PCR with two fragments: one fragment including positions +64 to +1493 of HVS A11 genome (GenBank accession number: X64346.1), and a second fragment including positions +1697 to +7249. The generated fragment, which lacks both HSUR1 and HSUR1 promoter sequence, was inserted between the *SalI* and *BamHI* sites of pBluescript II SK+ to generate the plasmid pBS-7.4ΔHSUR1. A unique *NcoI* site was generated by site-directed mutagenesis in pBS-7.4ΔHSUR1 [17] at a position corresponding to +3664 of HVS A11 genome to generate the plasmid pBS-7.4ΔHSUR1/Nco. A PCR amplificon containing the kanamycin resistance gene, the I-*SceI* restriction site, and a 50 bp duplication of HVS A11 genomic sequence was generated using the pEPkanS2 vector [18] (kindly provided by Karsten Tischer) and inserted into the *NcoI* site of pBS-7.4ΔHSUR1/Nco to generate the plasmid pBS-7.4ΔHSUR1/Nco/Kan. A PCR-amplified fragment encompassing positions +65 to +4637 of the HVS A11 genome was generated using pBS-7.4ΔHSUR1/Nco/Kan as template and recombined into the bacmid HVS-BAC-GFP [19] (kindly provided by Adrian Whitehouse) in GS1783 cells (a generous gift from Greg Smith) as previously described [20]. Recombinants were selected on chloramphenicol/kanamycin plates. The scarless removal of the kanamycin cassette was achieved by induction of I-*SceI* expression during the second Red-recombination step through arabinose induction, generating the bacmid HVS-BAC-GFP-ΔHSUR1. Positive clones were screened by PCR and analyzed by restriction endonuclease and sequencing.

### Cell and virus culture, transfections

Recombinant viruses were generated by transfection of bacmids HVS-BAC-GFP-WT and HVS-BAC-GFP-ΔHSUR1 into owl monkey (*Aotus trivirgatus*) kidney cells [American Type Culture Collection (ATCC), OMK-637-69] as previously described [7]. *In vitro* immortalization of marmoset T cells with these viruses was performed as described [7, 21]. Preservative-free heparinized marmoset blood samples from animals cj36416, cj38062, and cj38637 were obtained from the Southwest National Primate Research Center, aliquoted, and successfully immortalized in parallel with WT HVS or the mutant HVS lacking HSUR1 gene, generating cell lines cj36416-WT, cj36416-ΔHSUR1, cj38062-WT, cj38062-ΔHSUR1, cj38637-WT, and cj38637-ΔHSUR1. These cell lines were passed in parallel at the same time, and all experiments comparing them were performed using cells that have gone through the same number of passages. Ablation of HSUR1 from the HVS genome was confirmed by sequencing and Northern blotting (Supplementary Fig. S2A). The newly prepared WT and ΔHSUR1 cell lines as well as the cj319-WT cell line were grown as previously described [7]. 293T/17 (CRL 11268) and HeLa cells (CCL-2) were purchased from ATCC and grown as described [12]. MOLT-4 cells (obtained from ATCC, CRL1582) and human HuT78 Sézary Syndrome, cutaneous T lymphocytes (obtained from Vicente Planelles) were grown in RPMI-1640 medium supplemented with 10% FBS, antibiotics, glutamax, and sodium pyruvate. Cell lines were not authenticated. All cell lines were routinely tested for the presence of mycoplasma using the universal mycoplasma detection kit from ATCC.

For transient knockdown of HSUR1, 10 million cj319-WT marmoset T cells were nucleofected in a nucleofector™ 2b device as described previously [7] using 1 nmol of chimeric antisense oligonucleotide (ASO) (Integrated DNA Technologies) containing a backbone of phosphorothioate linkages and five 2′-*O*-methoxyethyl ribonucleotides at each end directed to either GFP (5′-mU*mC*mA*mC*mC*T*T*C*A*C*C*C*T*C*T*mC* mC*mA*mC*mU-3′) (control sample) or to HSUR1 (5′-mG*mG*mG*mU*mU*G*T*T*T*G*A*G*A*C*T*mU*mU* mU*mA*mG-3′), resulting in the specific degradation of HSUR1. Twenty-one hours after transfection, the cells were harvested, washed with phosphate buffered saline (PBS), and stored in TRIzol® at −80°C until used. To determine the abundance of HSUR1 target mRNAs by RT-qPCR, HuT78 cells were electroporated in quintuplicate using 2 million cells and either 5 µg of plasmids pBS-GFP-U1 or pBS-GFP-U1-HSUR1 per transfection (a total of 10 million cells electroporated per experiment). Cells were electroporated in 100 µl of buffer T by applying three 10 ms pulses of 1500 V each using the Neon™ transfection instrument (Invitrogen). Transfected cells were plated in 2 ml of complete media per well of a six-well plate, grown for 21 h at 37°C/5% CO_2_, harvested, and pooled. Cells were dissociated with StemPro Accutase® and suspended in 2 ml media containing 100 ng/ml of 2-(4-amidinophenyl)-1H-indole-6-carboxamidine (DAPI) before being fluorescence-activated cell sorting (FACS)-sorted for DAPI-negative, GFP-positive cells using an 85 µm nozzle and the 408 nm (violet 450/50 filter set) and 488 nm (blue 530/30 filter set) lasers. For luciferase reporter assays (see below), 2 million HuT78 cells constitutively expressing firefly luciferase reporters and *Renilla* luciferase were electroporated using 7.5 µg of either pBS-GFP-U1 or pBS-GFP-U1-HSUR1 (WT or bearing different mutations as indicated), plated in 2 ml of complete media, grown for 21 h plus at 37°C/5% CO_2_, and harvested. Cells were dissociated, suspended in 1 ml of DAPI-containing media, and FACS sorted for live, GFP-positive cells. To determine the effect of ZFP36L2 expression on firefly luciferase reporters in the presence of WT or mutant HSUR1, HuT78 cells were co-transfected with 7.5 µg of either pBS-GFP-U1 or pBS-GFP-U1-HSUR1 and 0.5 µg of a plasmid encoding mCherry and ZFP36L2 proteins. Transfected HuT78 cells were grown for 18–21 h after electroporation, dissociated, stained with DAPI, and FACS-sorted using an 85 µm nozzle and the violet 408 nm, blue 488 nm, and yellow–green 561 nm (610/20 bandpass filter set) lasers for live DAPI-negative and GFP- and mCherry-double-positive cells. Immediately after sorting, HuT78 cells were incubated for 1 h at 37°C/5% CO_2_, centrifuged at 1000 × *g* for 20 min, and then used in dual luciferase assays as described below. HeLa cells were transfected as previously described [12] with either Lipofectamine 2000 (Thermo Fisher Scientific) or Lipofectamine RNAiMax (Thermo Fisher Scientific) following the manufacturer’s instructions. Cells in 10 cm plates were transfected with 150 pmol of miRNA (control miRNA, miR-142-3p, miR-142-3pCC; Integrated DNA Technologies), 150 pmol of locked nucleic acid (LNA) inhibitor (control LNA or miR-27 LNA; miRCURY LNA^TM,^ Exiqon), and 10 µg of ZFP36L2-mCherry2, Control-mCherry2, or GFP-TIS11B (Addgene #121157). Cells in six-well plates were transfected with 25 pmol of miRNA or LNA miRNA inhibitors as described [12]. In all experiments, cells were harvested ∼24 h after transfection. miRCURY LNA^TM^ miRNA Inhibitor Negative Control A was used as Control LNA, and a miR-142-3p scrambled sequence (5′-AUGUUGUCCGUUAUUCAGUUAUG-3′) was used as Control miRNA.

All lentiviral transductions were performed in HuT78 or HeLa cells with supernatants generated as previously described [12]. Stable cell lines were generated by selection with either blasticidin for *Renilla* luciferase-expressing HuT78 cells or blasticidin and puromycin for *Renilla* and firefly luciferase-expressing HuT78 cells, or only puromycin for HSUR1- and mutant HSUR1-expressing HuT78 and HeLa cells. Stably transduced firefly- and *Renilla*-luciferase-expressing HuT78 cells (see below) were grown in complete medium plus 8 µg/ml of puromycin (InvivoGen) and 10 µg/ml of blasticidin (InvivoGen), whereas stably transduced HuT78 and HeLa cells expressing WT or mutant versions of HSUR1 were grown in complete medium plus 10 or 8 µg/ml of puromycin, respectively.

Cytotoxicity of marmoset cell lines was determined using the CytoTox-Glo™ reagent kit (Promega) on 1 × 10^5^ marmoset cells that were incubated for 24 h with 1 × 10^4^ human T lymphoblastoid MOLT-4 cells. Luminescence due to MOLT-4 cell cytotoxicity in the presence of a marmoset cell line was determined by subtracting the average luminescent signal of wells containing marmoset cells only from the luminescent signal measured in wells containing both MOLT-4 and marmoset cells. Total luminescence in MOLT-4 cells was determined from the MOLT-4-only wells treated with 50 µl of lysis reagent containing digitonin (100% cytotoxicity). The relative cytotoxicity of MOLT-4 cells in the presence of marmoset cells (expressed as a percentage) was calculated by dividing the luminescence of MOLT-4 cells measured in the presence of marmoset cells by the total luminescent signal of MOLT-4 cells treated with digitonin.

### iRICC of HSUR1

iRICC was performed as described [8] with a few modifications as follows. Each replicate sample was mixed with 160 pmoles of DNA ASO to the entire HSUR1 sequence (ANTI-HSUR1protect: 5′-TGGTACCGGTCATCATATTTACACCCAGTACC TACAAAAATTGGTCAGCAAGTAACGGGTTGTTTGAG ACTTTTAGTTTAGGTTATCACAGATTTA AGTTCCAGTAAACATTACTAAGAAATAAAT AAATAAATATGTAGTGT-3′; Integrated DNA Technologies), for digestions with S1 nuclease and RNase H. Samples were annealed to 100 pmoles of biotinylated HSUR1 RNA ASO (b-H1rASO: 5′-/52-Bio/TTAArGrGrUrCrArGrCrArArGrUrA rArCrGrGrGrUrUrGrUrUrUrGrArGrArCrUrUrUrUr ArGrUrUrUrArGrGrUrUrArUrCrArCrArG/3SpC3/-3′; Integrated DNA Technologies) for capture of HSUR1 crosslinked to target RNAs as described [8]. Washes after captures were performed at 58°C. Library preparation and high-throughput sequencing were performed as previously described [8].

Samples for high-throughput sequencing for all three iRICC experiments were prepared in parallel from 8 µl of RNA for each sample using the SMARTer Stranded Total RNA-seq Kit v2—Pico Input Mammalian (Takara Bio USA) following the user manual, but with a few modifications as previously described [8]. HiSeq 125 cycle paired-end sequencing was performed by the High Throughput Genomics Core Facility at the Huntsman Cancer Institute (University of Utah) on an Illumina HiSeq 2500 instrument.

Two approaches were taken to identify potential HSUR1 target mRNAs. The first followed a standard bulk RNA-seq differential expression analysis. This entailed utilizing STAR (v2.5.3a) to create an index using a combined reference of *Callithrix jacchus* (v3.2.1, Ensemble v91) with saimiriine herpesvirus 2 (Genbank ID: X64346.1). STAR alignments were run on reads trimmed to remove the first three base pairs at the start of the reads and the ends of reads beginning at poly(A) stretches of 10 bp or longer as previously described [8]. Reads shorter than 10 bp after processing were also removed from the analysis [8]. The Subread ‘featureCount’ command was used to generate alignment counts for each gene in the annotation file, only counting reads on the same strand as the annotation. DESeq2 (v1.14.1) was used to normalize gene counts across the samples and determine differential expression between the HSUR1 and control samples. The *Callithrix jacchus* genome, the alignment depth BigWig (.bw), and a region file of differentially expressed HSUR1 targets identified by DESeq2 (FDR < 0.05, log_2_Rto > 0.585) were loaded into the IGV browser (v.2.4.16) and visually inspected. This revealed that some transcripts adjacent to the analyzed genes also contained discrete peaks of enriched reads in the HSUR1 samples relative to the controls and had been omitted from the list of potential HSUR1 targets.

The second method for identifying HSUR1 targets utilized a sliding window, enriched region approach originally developed for ChIP-seq analysis [22], with the three iRICC replicates. STAR alignments were run using the combine index as described earlier but just using the first read to maximize small read alignment. The six alignment files were filtered for those that uniquely mapped (MQ ≥ 13) and were converted to center position PointData with the USeq SamParser. Each replica pair was window scanned using the USeq ScanSeqs application, and overlapping windows passing thresholds (Q-value false discovery rate ≥ 0.05, and log_2_Rto ≥ 1) were merged using the USeq EnrichedRegionMaker. The USeq CompareIntersectingRegions application was run to identify enriched regions found by two or three of the replicates. Lastly, the USeq AnnotateBedWithGenes app was used to annotate the enriched regions with intersecting marmoset gene names.

Combining the two approaches generated a list of over 1000 potential HSUR1 targets. This list was curated to identify unannotated genes as well as unidentified gene regions in the IGV browser by performing BLAST and confirming the identification in Ensembl as well as the Gene database of the National Library of Medicine. Genes that remained unidentified after these analyses were eliminated from the list of potential HSUR1 targets. The coordinates of genes conforming to these criteria were inserted along with the assembled marmoset genome into the IGV 2.4.16 genome browser to visually inspect the peaks of aligned reads using both read1 and read2 across each gene and across all experiments. We then eliminated from further analyses pseudogenes, genes that showed high background in control samples, genes that lacked clear and discrete peaks of aligned reads identifiable in all three experiments, and RNAs that had peaks of aligned reads with inconsistent boundaries that did not allow for unambiguous determination of crosslinking sites. This resulted in 867 HSUR1 targets listed in Supplementary Table S1, of which 213 targets with peaks at either the 3'UTR, CDS/3’UTR boundaries, or both were subjected to further analyses to determine potential base-pairing with HSUR1 (Supplementary Table S1). These target mRNAs exhibited peaks with abrupt edges or a sharp drop of aligned reads in either read1, read2, or both across all three experiments, a characteristic that was used as the primary indicator of a potential site of crosslinking between target mRNAs and HSUR1. Finally, we used the Ensembl database, the Gene database of the National Library of Medicine, and the UCSC Genome Browser for the white-tufted-ear marmoset to obtain 3’UTR sequences of HSUR1 targets that were unannotated in the IGV genome browser but that clearly showed peaks of aligned reads within this region.

To determine the 3′UTR sequences that might be involved in base-pairing with HSUR1, an initial window of 40 nucleotides centered at the potential site of crosslinking (sharp drop of sequencing reads) was entered into the RNAcofold web server from the ViennaRNA Package 2.0 at the Institute of Theoretical Chemistry at the University of Vienna along with the full-length sequence of HSUR1 using default parameters as previously described [8]. The window of sequence in the target mRNA was sequentially increased to 60 and 80 nt until the results of two analyses (i.e. 40 and 60 nt or 60 and 80 nt) yielded similar binding patterns between the target mRNA sequence and HSUR1. The minimal sequences mediating the interaction of target mRNAs and HSUR1 were then determined in RNAcofold after trimming the sequences not predicted to be involved in contiguous base-pairing between the target mRNA and HSUR1 as described [8]. The analysis was also used to obtain an estimated Δ*G* of binding and minimal free energy for the interaction between HSUR1 and HSUR1 binding sites on target mRNAs. The predicted interactions between HSUR1 binding sites in target transcripts and HSUR1 sequences were visualized using the RNAfold web server from the ViennaRNA Package 2.0 as previously described [8] by inserting 5 nt (e.g. AAAAA or UUUUU) between the two interacting sequences and folding the sequence onto itself, ensuring that the inserted nucleotides formed a connecting loop. This confirmed that potentially crosslinked pyrimidines were present at the end of helical runs or at G-U wobble base pairs. A final list of 313 high confidence HSUR1 binding sites on target genes located at 3’UTRs or CDS/3’UTR boundaries and showing their binding pattern to HSUR1 is shown in Supplementary Table S1.

### RNA sequencing

Marmoset cj38637-WT and cj38637-ΔHSUR1 T cells were grown in quintuplicate, harvested, and counted. Twenty million cells in each replicate sample were treated with StemPro Accutase® (Gibco), centrifuged, and suspended in 2 ml of complete media containing 100 ng/ml of DAPI. Cells were FACS-sorted at the University of Utah Flow Cytometry Core on a BD FACSAria-II 4-laser cell sorter (BD Biosciences) equipped with BD FACSDiva v8.0 software using a 70 µm nozzle and a 408 nm (violet 450/50 filter set) laser. Five million live DAPI-negative cells were collected per sample and stored in TRIzol® at −80°C until used. Total RNA was purified according to the manufacturer’s instructions using 30 µg of glycogen during the isopropanol precipitation step. The RNA pellets were washed with 70% ethanol and air-dried. The RNA pellets were then suspended in 85 µl of water, 10 µl of 10X DNase I buffer (New England Biolabs), and 5 µl of RNase-free DNase I (New England Biolabs). Samples were incubated at 37°C for 30 min and RNA was purified on RNA clean & concentrator™-5 columns (Zymo Research) using RNA binding buffer according to the manufacturer’s protocol (Zymo Research) and eluted in 15 µl of water. RNA sequencing libraries were prepared using the NEBNext® Ultra II™ Directional RNA Library Prep with poly(A)+ mRNA isolation kit (New England Biolabs). Sequencing libraries were chemically denatured in preparation for sequencing. Following transfer of the denatured samples to an Illumina NovaSeq X instrument, a 151 × 151 cycle paired-end sequence run was performed using a NovaSeq X Series 10B Reagent Kit (20085594).

### RNA-seq analyses

The RNA-seq reads were aligned to the CalJac4 genome assembly and its associated RefSeq transcriptomic annotations using STAR (v2.5.3a). Subsequently, gene quantification was performed using RSEM (v1.3.0.0), and statistics for differential gene expression were performed using limma (v3.58.1). CIBERSORTx analysis was performed using the online tool (https://cibersortx.stanford.edu), using full gene expression matrices as input after conversion of marmoset gene names to their human equivalent. For splicing analysis, rMATS (v4.2.2) was used to determine differentially spliced events, with significant events having an adjusted *P*-value ≤ .05 and an absolute change in percentage spliced in (|ΔPSI|) ≥ 5%. The homology of differentially spliced exons to their human counterparts was assessed by comparing CalJac4 sequences to corresponding loci in Hg38, with exons pairs that have the exact same length and high sequence similarity being considered highly orthologous.

### Gene ontology-term enrichment analyses

The complete high-confidence list of HSUR1 target mRNAs generated by iRICC-seq (Supplementary Table S1) was copied into the ShinyGO 0.80 gene ontology (GO) suite (http://bioinformatics.sdstate.edu/go/) and analyzed using the *Callithrix jacchus* (white-tufted-ear marmoset) mCaloJac1.pat.X gene assembly from Ensembl, a default FDR cutoff of 0.05, and a background list comprised of all the genes identified by iRICC sequencing. GO and Kyoto Encyclopedia of Genes and Genomes (KEGG) pathways that showed an enrichment *P*-value ≤ .05 are shown in Supplementary Table S2, whereas selected significant pathways are shown in Fig. 6A. GO analyses of differentially expressed genes in cj38637-ΔHSUR1 cells versus cj38637-WT cells (Supplementary Table S8) was performed in a similar manner using the entire list of genes for which expression changed at least 1.5-fold, as determined by RNA-seq, against a background list of all the marmoset genes sequenced. Select pathways that showed an enrichment *P*-value < .005 are shown in Supplementary Fig. S9.

### Flow cytometry

Antibodies used in flow cytometry as well as their sources, catalog numbers, and dilution are listed in Supplementary Table S7. Marmoset cj36416-WT, cj36416-ΔHSUR1, cj38062-WT, cj38062-ΔHSUR1, cj38637-WT, cj38637-ΔHSUR1, HuT78 cells, and HuT78 cells expressing HSUR1 were harvested and suspended in complete media at a concentration of 5 million cells per ml. For each antibody assay, 200 µl of each marmoset cell line and 100 µl of either WT or HSUR1-expressing HuT78 cells were added in triplicate per well of a 96-well plate. Cells were blocked with 100 µl of eBioscience™ Flow Cytometry Staining Buffer (Invitrogen) by incubating on ice for 15 min. Cells were stained with 100 µl of staining buffer containing fluorescent antibodies to either CD28, CD4, CD3, CD8a, CD16, CD56, or CD57 and incubated on ice protected from light for 40 min. Cells were washed three times with 100 µl of staining buffer and suspended in 100 µl of staining buffer containing 100 ng/ml of DAPI, except for the cells stained with BV421 (CD57), which were suspended in 100 µl of staining buffer containing 10 ng/ml of propidium iodide. To detect CD25, marmoset cells were incubated with 100 µl of staining buffer containing unconjugated rabbit polyclonal antibody to human CD25 on ice for 40 min. Anti-CD25 was then detected by incubating the cells with 100 µl of staining buffer containing R-PE-conjugated goat anti-rabbit IgG (H + L) on ice protected from light for 30 min. The cells were washed and suspended in staining buffer with DAPI as described earlier. Data were acquired on a BD FACSCanto (BD Biosciences) flow cytometer equipped with BD FACSDiva 6.1.3 software using the 405 nm violet (450/50 bandpass filter set), 488 nm blue (530/30 bandpass filter set, 670 long pass filter), 561 nm yellow–green (585/15 bandpass filter set), and 640 nm red (670/30 bandpass filter set) lasers.

### Activation of marmoset cells and densitometry of IFN-γ

Four million cj36416-WT, cj36416-ΔHSUR1, cj38062-WT, cj38062-ΔHSUR1, cj38637-WT, or cj38637-ΔHSUR1 were seeded in triplicate in 2 ml of media containing 1X eBioscience™ cell stimulation cocktail [81 nM phorbol 12-myristate 13-acetate (PMA), 1.34 µM ionomycin, 10.6 µM brefeldin A and 2 µM monensin; Thermo Fisher Scientific] and incubated at 37°C/5% CO_2_ for 5 h. Cells were harvested, and total extracts were prepared and analyzed by immunoblotting as described below. Blots were analyzed by densitometry using ImageJ, with the values corresponding to IFN-γ in each lane being divided by the corresponding values of histone deacetylase 1 (HDAC1, used as a loading control; see below). Abundance of IFN-γ in extracts from cells infected with HVS lacking HSUR1 was expressed relative to the abundance of IFN-γ in extracts from cells infected with WT virus set as 1.0.

### Immunoblotting

Antibodies, sources, as well as dilutions used for immunoblotting experiments are listed in Supplementary Table S7. Marmoset cj38637-WT and cj38637-ΔHSUR1 cells were collected, stained with DAPI, and FACS-sorted as described earlier. Total cell lysates were prepared in Glo Lysis Buffer (Promega) containing cOmplete™ Protease Inhibitors (one-half tablet per 5 ml of lysis buffer, Roche), 1 mM phenylmethanesulfonyl fluoride (Sigma), and 1X Halt™ Phosphatase Inhibitor Cocktail (Thermo Fisher Scientific). The protein concentration of the lysates was determined with the Pierce™ Bradford Plus Protein Assay reagent (Thermo Fisher Scientific). Lysates were boiled with 1× sodium dodecyl sulfate–polyacrylamide gel electrophoresis (SDS–PAGE) sample buffer, and 20 µg total protein was separated on 4%–20% gradient gels (Bio-Rad) using high Tris running buffer (50 mM Tris base, 193 mM glycine, 0.1% SDS). Proteins were transferred onto nitrocellulose membranes, probed with antibodies to ADAR1, HNRNPA2B1, PIP4K2B, UBA2, or JARID2, as indicated, and visualized with IR-Dye® 680RD goat anti-rabbit IgG secondary antibodies and an Odyssey fluorescent scanner (LI-COR). In all cases, cofilin was used as loading control and visualized with IR-Dye® 680RD goat anti-rabbit IgG secondary antibodies as described above. To detect IFN-γ, lysates from cj36416-WT, cj36416-ΔHSUR1, cj38062-WT, cj38062-ΔHSUR1, cj38637-WT, and cj38637-ΔHSUR1 cells stimulated with PMA and ionomycin were prepared in Glo Lysis Buffer containing protein and phosphatase inhibitors, run on SDS–PAGE gradient gels, and transferred to nitrocellulose membranes as described earlier. The membranes were then incubated sequentially with rabbit monoclonal antibodies to IFN-γ and HDAC1 and visualized each time with IR-Dye® 680RD goat anti-rabbit IgG secondary antibodies. To blot for TCF1, T-bet, RUNX3, and CD44, marmoset cj36416-WT, cj36416-ΔHSUR1, cj38062-WT, cj38062-ΔHSUR1, cj38637-WT, and cj38637-ΔHSUR1 cells were harvested, treated with StemPro Accutase®, DAPI-stained, and FACS-sorted. Two million live cells were collected in 5 ml of complete RPMI medium, centrifuged, and resuspended in 200 µl of 1× SDS–PAGE sample buffer. Twenty-microliter samples were separated on 4%–20% gradient gels, transferred to nitrocellulose membranes, and probed, as indicated, with polyclonal antibodies to TCF1, T-bet or RUNX3, or rabbit monoclonal antibodies to CD44. Detection of ARE-BP in cell extracts shown in Supplementary Figs S4B, C and S5A was performed with rabbit monoclonal antibodies to TTP, ZFP36L1, ZFP36L2, or KH-type Splicing Regulatory Protein (KHSRP). Rabbit polyclonal antibodies were used to detect HuR and hnRNP D (Supplementary Fig. S5A). In all cases, rabbit antibodies were visualized with fluorescent IR-Dye® 680RD goat anti-rabbit IgG secondary antibodies as described earlier.

For ARE-BP recruitment experiments, 20 µl of the input and IP fractions in SDS–PAGE buffer from cj38637-WT and cj38637-ΔHSUR1 extracts subjected to immunoprecipitation with anti-ARE-BP antibodies (see below) were separated on 4%–20% polyacrylamide gradient gels as described earlier. After transfer onto nitrocellulose, the membranes were immunoblotted with rabbit polyclonal antibody to HuR (Supplementary Fig. S5D) (same antibody used for immunoprecipitation) or mouse monoclonal antibodies to hnRNP D (Supplementary Fig. S5E) or KHSRP (Fig. 5E). HuR was visualized as described earlier, whereas hnRNPD was visualized with IRDye® 680RD donkey anti-mouse IgG, and KHSRP was visualized with IRDye® 680RD goat anti-mouse IgM secondary antibodies.

### Luciferase reporter assays

Thirty thousand DAPI-negative and GFP-single positive or DAPI-negative and GFP- and mCherry-double-positive HuT78 cells were FACS-sorted into Eppendorf microcentrifuge tubes containing 0.5 ml of complete culture medium and recovered by centrifugation. Media was removed by aspiration, and the cell pellet was lysed with 50 µl of passive lysis buffer (Promega) for 20 min at room temperature with occasional vortexing. Ten microliters of each sample were plated in triplicate per well of a 96-well plate and analyzed for firefly and *Renilla* luciferase activities as previously described [8]. Normalized light units (NLU) were obtained by dividing the firefly luciferase relative light units (RLU) by the corresponding *Renilla* RLU of each well. The relative luciferase activity was then calculated by dividing the NLU of each sample by the average NLU of the control sample set as 1.0.

### Quantitative real-time polymerase chain reaction

Total RNA was purified from HeLa, HuT78, or from 5 million FACS-sorted live cj319-WT, cj38637-WT, or cj38637-ΔHSUR1 cells stored in TRIzol®. cDNA was synthesized in 20 µl reactions from 900 ng of DNase I-treated total RNA using the High-Capacity cDNA Reverse Transcription Kit with MultiScribe Reverse Transcriptase and random primers (Applied Biosystems). Real-time PCR was performed in 8 µl reactions using primers (see Supplementary Table S5) at 0.5 µM, cDNA diluted at a ratio of 1:10 (with the exception of ribosomal 18 S, for which the cDNAs were diluted 1:50 000), and PerfeCTa® SYBR® Green FastMix® (Quantabio) in a Bio-Rad CFX Opus 384 Light Cycler (95°C for 5 min, 1 cycle; 95°C for 10 s, 60°C for 10 s, and 72°C for 10 s, 55 cycles followed by a melting curve of the reaction product from 65°C to 95°C with a ramp rate of 0.5°C per second, 5 s plate read). qPCR primers were designed using Primer3Plus (https://www.primer3plus.com/index.html) and tested using cDNA prepared from 900 ng of DNase I-treated total RNA from each cell line in 20 µl reactions and diluted at ratios 1:5–1:50 000. All reactions were performed in triplicate in each independent experiment. *C*_t_ values were determined using the instrument’s automated functions for baseline and threshold setting and *C*_t_ determination. Relative mRNA abundance of HSUR1 targets were calculated using the 2^−ΔΔ^*^C^*_t_ method [23], dividing the 2^−Δ^*^C^*_t_ of the HSUR1 target in the treated sample by the 2^−Δ^*^C^*_t_ of the HSUR1 target in the control sample (empty vector or ΔHSUR1). For normalization, the *C*_t_ values of 18S ribosomal RNA were subtracted from the *C*_t_ values of HSUR1 targets to calculate Δ*C*_t_.

### Apoptosis assays

Growing cultures of cj36416-WT, cj36416-ΔHSUR1, cj38062-WT, cj38062-ΔHSUR1, cj38637-WT, and cj38637-ΔHSUR1 were cleaned of cell debris on Lymphoprep™ cushions according to the manufacturer’s instructions (STEMCELL Technologies), seeded in T25 flasks in 5 ml of media at a density of 0.5 × 10^6^ cells/ml, and grown for 14 days. On day 15, the cells were collected, counted, washed in ice-cold PBS, centrifuged, and suspended at a density of 10^6^ cells/ml in 100 µl annexin-binding buffer (10 mM HEPES, pH 7.4, 140 mM NaCl, and 2.5 mM CaCl_2_). Cells were stained with annexin V-Alexa Fluor® 647 conjugate (1:20 dilution, Life Technologies) and 1 µg/ml of propidium iodide (Invitrogen) as described [7]. Data were acquired on a BD FACSCanto (BD Biosciences) flow cytometer using the 488 nm blue (685/40 bandpass filter set) and 640 nm red (670/30 bandpass filter set) lasers as previously described [7]. Alternatively, 40 000 live DAPI-negative cj38637-WT and cj38637-ΔHSUR1 cells were FACS-sorted directly per well of a 96-well plate. The cells were then incubated with 100 ng/ml recombinant soluble Fas ligand (PeproTech) for 21 h at 37°C. Caspase activity was measured with the Caspase-Glo™ 3/7 reagent according to the manufacturer’s instructions (Promega). We also measured caspase 3/7 activity in HuT78 cells expressing HSUR1. Stable HSUR1-expressing HuT78 cells were prepared by lentiviral transduction and selection with 8 µg/ml of puromycin as described earlier. Control and HuT78-HSUR1 cells were suspended in media at a concentration of 400 000 cells/ml, and 100 µl were seeded per well of a 96-well plate. Soluble Fas ligand was added to each well to a final concentration of 100 ng/ml and incubated for 21 h at 37°C. Caspase 3/7 activity was determined with Caspase-Glo™ 3/7 reagent.

### Immunoprecipitations

Seventy million cj38637-WT and cj38637-ΔHSUR1 marmoset cells were collected, washed with PBS, and suspended in 0.3 ml of NET2 buffer (100 mM Tris–HCl, pH 7.5, 150 mM NaCl, 0.05% Nonidet P-40 substitute (Roche), 10 µl of SUPERase-in™ RNase inhibitor (Invitrogen), cOmplete™ Protease Inhibitor Cocktail (one-half tablet for every 5 ml of NET2 buffer), 1 mM PMSF, and 1X Halt™ Phosphatase Inhibitor Cocktail). Cells were sonicated on ice using a Branson Sonifier 450 sonicator on output setting #3 and 10 cycles at 80% duty to lyse the cells. Fifteen microliters from each sample were saved as input fractions and stored in 1 ml of TRIzol® at −80°C. Alternatively, 15 µl of each sample were mixed with 15 µl of water and 30 µl of 2× SDS sample buffer and heated to 95°C for 5 min. Fifty microliters of protein A Dynabeads™ (Invitrogen) prebound to antibodies (see below) were added to each sample and incubated with continuous rotation at 4°C for 6 h. Samples were placed on the magnetic rack, and the supernatants were aspirated and discarded. The beads were washed three times with NET2 buffer without inhibitors and transferred after the third wash to a fresh Eppendorf tube and placed on the magnetic rack. The supernatant was aspirated, and 1 ml of TRIzol® was added to each sample and stored at −80°C until used. Alternatively, the beads were suspended in 60 µl of 1× SDS sample buffer, heated at 95°C for 5 min, and stored at −80°C until used. To prepare the protein A Dynabeads™ for immunoprecipitation, 100 µl of beads were washed with 1 ml of NET2 buffer without inhibitors, placed on the magnetic rack, and the supernatant aspirated. The beads were suspended in 1 ml of NET2 buffer without inhibitors and mixed with either 4.5 µg of rabbit polyclonal antibody to HuR, 20 µg of rabbit polyclonal antibody to hnRNP D, 2 µg of rabbit monoclonal antibody to KHSRP, or 40 µl of normal rabbit serum (Invitrogen) and incubated with continuous rotation at 4°C for 2 h. After incubation, the tubes were placed on the magnetic rack and the supernatant was aspirated. The beads were then suspended in 100 µl of NET2 buffer plus inhibitors and 50 µl were then added, respectively, to the extracts from cj38637-WT cells and cj38637-ΔHSUR1 cells. To test whether HSUR1 bound to target mRNAs could recruit ZFP36L1, control HeLa cells or HeLa-H1 cells were seeded on 10 cm tissue culture dishes at a density of 6 × 10^6^ cells per plate. The following day, the cells were transfected with 10 µg of a plasmid encoding GFP fused to ZFP36L1 (GFP-TIS11B), 150 pmol of miR-142-3p, and 50 µl of Lipofectamine 2000 per plate according to the manufacturer’s protocol (Thermo Fisher). Cells were harvested 21 h after transfection and extracts were prepared as described earlier. Extracts were incubated with 5 µg of polyclonal antibody to GFP prebound to protein A Dynabeads™ per sample as described earlier. Recruitment of ZFP36L2 to WT HSUR1 or mutated versions of HSUR1 thereof bound to target mRNAs was interrogated by co-transfecting HeLa cells as described earlier with miR-142-3p and mCherry2-ZFP36L2. Cell extracts were prepared as described and incubated with 5 µg of a mouse monoclonal antibody to the V5 tag prebound to protein A Dynabeads™.

### Analysis of mRNAs immunoprecipitated with ARE-binding proteins

To determine the relative abundance of HSUR1 target mRNAs in the immunoprecipitates (IP) from cj38637-WT and cj38637-ΔHSUR1 cells, or in the IP fractions of control HeLa cells and HeLa cells expressing either WT HSUR1 or its mutant versions, RNA in TRIzol® from the input and IP fractions was purified according to the manufacturer’s protocol, treated with RNase-free DNase-I, and purified on RNA Clean & Concentrator™-5 columns (Zymo Research). The RNA was eluted from the columns with 20 µl of water. cDNA was synthesized by assembling 20 µl reactions containing 2.5 µl of each input fraction plus 11.7 µl of water, or 14.2 µl of each IP fraction plus, in all cases, 2 µl of 10× reaction buffer, 2 µl of random primers, 0.8 µl of dNTPs, and 1 µl of MultiScribe Reverse Transcriptase (Applied Biosystems). Real-time PCR was performed in 8 µl reactions as described earlier except that samples were normalized by subtracting the average *C*_t_ value for each target in the input fractions (to calculate Δ*C*_t_). Enrichment of HSUR1 target mRNAs in the IP fractions from cj38637-WT cell extracts relative to those from cj38637-ΔHSUR1 cell extracts was calculated by dividing the 2^−Δ^*^C^*_t_ of the HSUR1 target in the WT samples by the 2^−Δ^*^C^*_t_ of the target in the ΔHSUR1 samples (set as 1.0). Enrichment of HSUR1 target mRNAs in the anti-V5 or anti-GFP IP fractions of HeLa-H1 cells relative to those from transfected control HeLa cells was calculated by subtracting the amount of each target mRNA present in the input fractions and then dividing the 2^−Δ^*^C^*_t_ of the HSUR1 target in the samples from HeLa-H1 cells by the 2^−Δ^*^C^*_t_ of the target in the sample from control HeLa cells (set as 1.0).

### Statistical analyses

**Figure 2.**
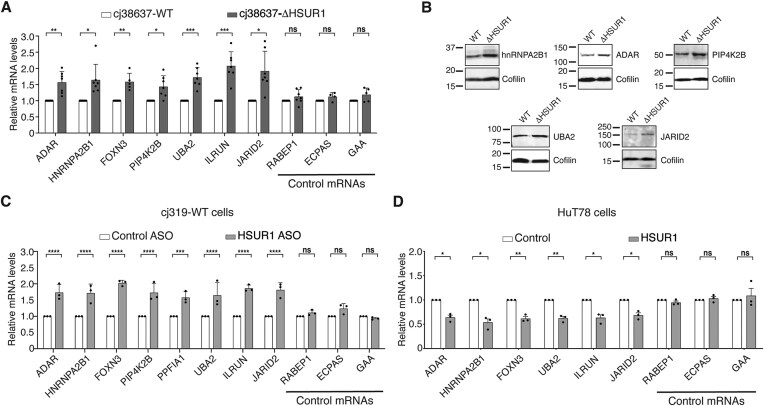
HSUR1 represses target mRNAs through their 3′UTRs. (**A**) Quantitative real-time polymerase chain reaction (qRT-PCR) analysis of representative HSUR1 target mRNAs bound only at the 3′UTR (ADAR, HNRNPA2B1, FOXN3, PIP4K2B, UBA2, ILRUN, and JARID2) and control mRNAs (RABEP1, ECPAS, and GAA) in cj38637-WT and cj38637-ΔHSUR1 cells. (**B**) Western blot of HSUR1 targets in cj38637-WT and cj38637-ΔHSUR1 cells. Cofilin provides a loading control. (**C**) As in panel (A) in cj319-WT cells transiently transfected with control or HSUR1 ASO. (**D**) As in panel (A) in HuT78 cells sorted by FACS for GFP after transient transfection with either a plasmid expressing GFP and the U1 snRNA promoter (Control) or a plasmid expressing GFP and HSUR1 under the U1 promoter (HSUR1). *P < .05; **P < .01; ***P < .001; ****P < .0001.

**Figure 3.**
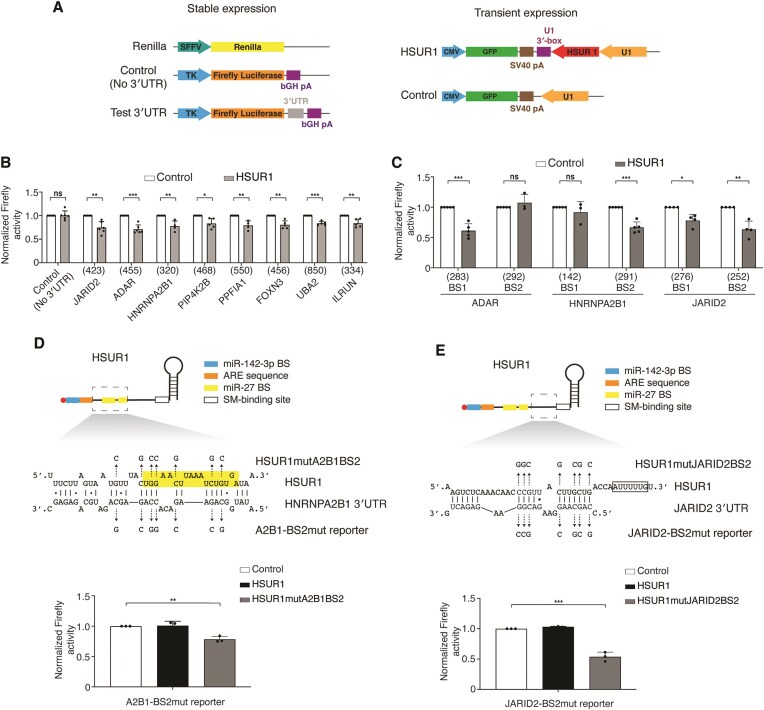
HSUR1 regulates target mRNAs through sequences defined by iRICC-seq. (**A**) Schematic of constructs used for stable expression of luciferase reporters and transient expression of HSUR1. *Renilla* luciferase was expressed from the spleen focus-forming virus promoter, and firefly luciferase from the thymidine kinase (TK) promoter, either without a 3′UTR (Control) or fused to partial 3′UTRs of HSUR1 target mRNAs. (**B**) Luciferase assays validating HSUR1 binding sites. Normalized luciferase levels in FACS-sorted, GFP-positive HuT78 cells stably expressing either control or 3′UTR-containing luciferase reporters after transient transfection with the indicated plasmids. The sizes of 3′UTR fragments (in base pairs) are indicated between parentheses. (**C**) Same as in panel (B) but using reporters containing individual HSUR1 binding sites. (**D-E**) Same as in panel (C) for HNRNPA2B1 (A2B1)-BS2mut (D) and JARID2-BS2mut (E) reporters. *P < .05; **P < .01; ***P < .001.

**Figure 4.**
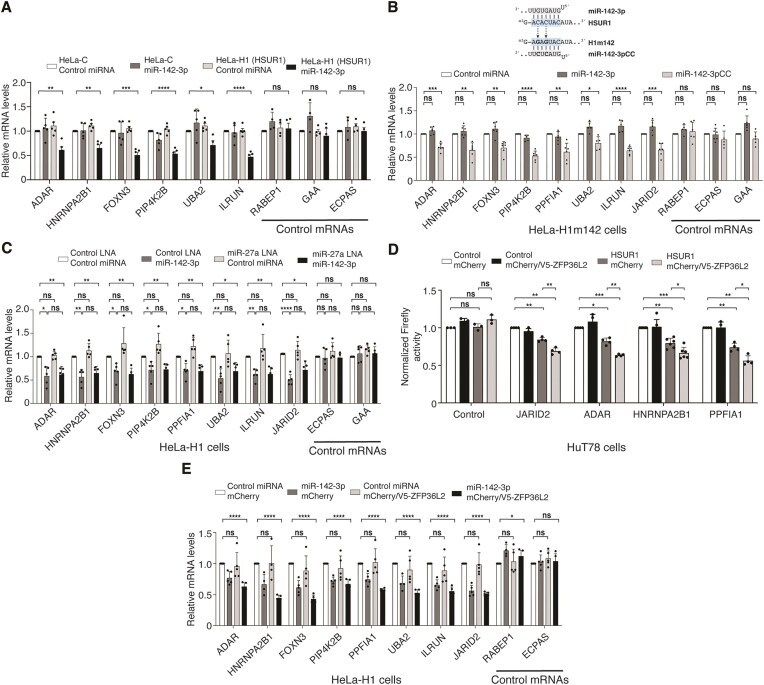
HSUR1 utilizes miR-142-3p and ARE-BPs to regulate target mRNAs. (**A**) qRT-PCR analyses of HSUR1 target mRNAs in control HeLa cells (HeLa-C) or HeLa cells stably expressing HSUR1 (HeLa-H1) after transient transfection with either a scrambled control miRNA or miR-142-3p. (**B**) Same as in panel (A), using HeLa cells expressing a mutant version of HSUR1 (HeLa-H1m142) containing point mutations in the miR-142-3p binding site (sequence shown above). Cells were transfected with control miRNA, WT miR-142-3p, or a compensatory mutant (miR-142-3pCC) designed to restore base pairing to H1m142. (**C**) Same as in panel (A) in HeLa-H1 cells co-transfected with control or miR-142-3p and either a control LNA inhibitor or an LNA targeting miR-27. (**D**) Dual-fluorescence reporter assay in cells sorted for GFP and mCherry expression following co-transfection with plasmids encoding mCherry alone (mCherry) or mCherry plus V5-tagged ZFP36L2 (mCherry/V5-ZFP36L2), together with a control GFP construct (U1 promoter) or GFP with HSUR1 under the U1 promoter. (**E**) Same as in panel (C) after co-transfection with mCherry or mCherry/V5-ZFP36L2 expression plasmids and either control miRNA or miR-142-3p. *P < .05; **P < .01; ***P < .001; ****P < .0001.

**Figure 5.**
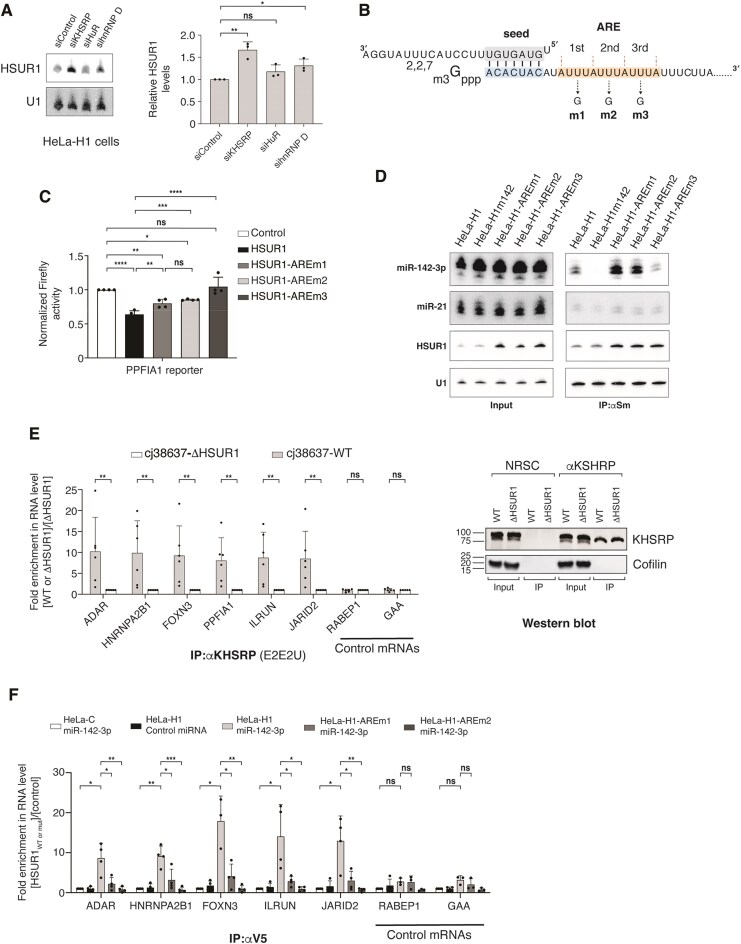
Dual role of the HSUR1 ARE in RNA stability and target repression. (**A**) Northern blot showing HSUR1 levels in HeLa-H1 cells transfected with control siRNA or siRNAs targeting KHSRP, HuR, or hnRNP D. U1 serves as a loading control. Quantification of three independent experiments is shown on the right; U1 snRNA signal was used for normalization. (**B**) Sequence of the HSUR1 5′ end and miR-142-3p showing mutations disrupting the first (m1), second (m2), or third (m3) AUUUA motif. (**C**) Reporter assay using HuT78 cells expressing the luciferase-PPFIA1 3′UTR reporter and co-transfected with mCherry/V5-ZFP36L2 and WT or mutant HSUR1 constructs as in panel (B). (**D**) Co-immunoprecipitation of miRNAs with αSm antibodies from HeLa-H1, HeLa-H1m142, and HeLa-H1-AREm1–m3 cells transfected with miR-142-3p. Northern blot probed for miRNAs, HSUR1, and U1 (immunoprecipitation control). Input, 5%; αSm IP, 100%. (**E**) Enrichment of HSUR1 target and control mRNAs in KHSRP immunoprecipitates from cj38637-WT and cj38637-ΔHSUR1 cells. Western blots on the right show IP efficiency using normal rabbit serum control and αKHSRP antibodies (E2E2U). Input, 5%; IP, 100%. (**F**) Same as in panel (E) using V5 immunoprecipitates from HeLa cells co-transfected with mCherry/V5-ZFP36L2 and either control or miR-142-3p.

**Figure 6.**
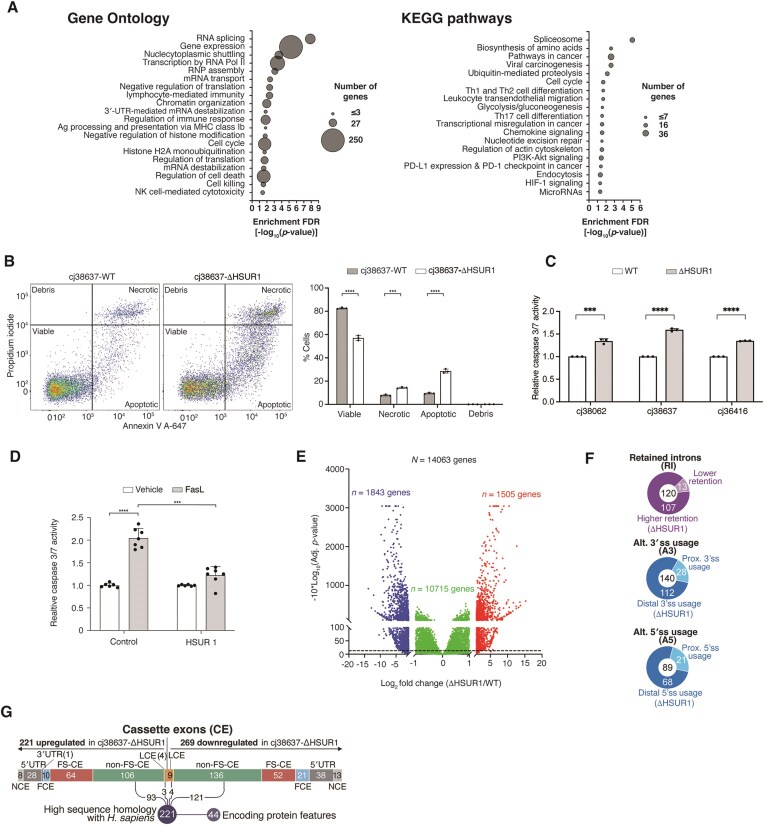
HSUR1 reprograms host gene expression and promotes cell survival. (**A**) GO and KEGG pathway analyses showing selected categories significantly enriched among HSUR1 target mRNAs. (**B**) Flow cytometry of cj38637-WT and cj38637-ΔHSUR1 cells stained with Annexin V–Alexa Fluor 647 and propidium iodide; quantification from three independent experiments shown on the right. (**C**) Caspase-3/7 activity in WT and ΔHSUR1 cells measured by Caspase-Glo 3/7 assay after FasL treatment. (**D**) Same as in panel (C), using HuT78 cells transduced with lentiviral vectors expressing GFP (control) or HSUR1 and treated with vehicle or FasL. (**E**) Volcano plot of differentially expressed transcripts in cj38637-ΔHSUR1 versus cj38637-WT cells. *Y* axis shows two different scales (separated by a break), with significant adjusted *P*-values shown above the dashed lines. (**F**) Summary of changes in intron retention and alternative splice site usage. (**G**) Summary of differentially spliced cassette exons. NCE, noncoding transcript exon; 5′UTR, 5′ untranslated region exon; FCE, first coding exon; FS-CE, frameshifting internal coding exon; non-FS-CE, non-frameshifting internal coding exon; LCE, last coding exon. *** *P* < .001, **** *P* < .0001.

**Figure 7.**
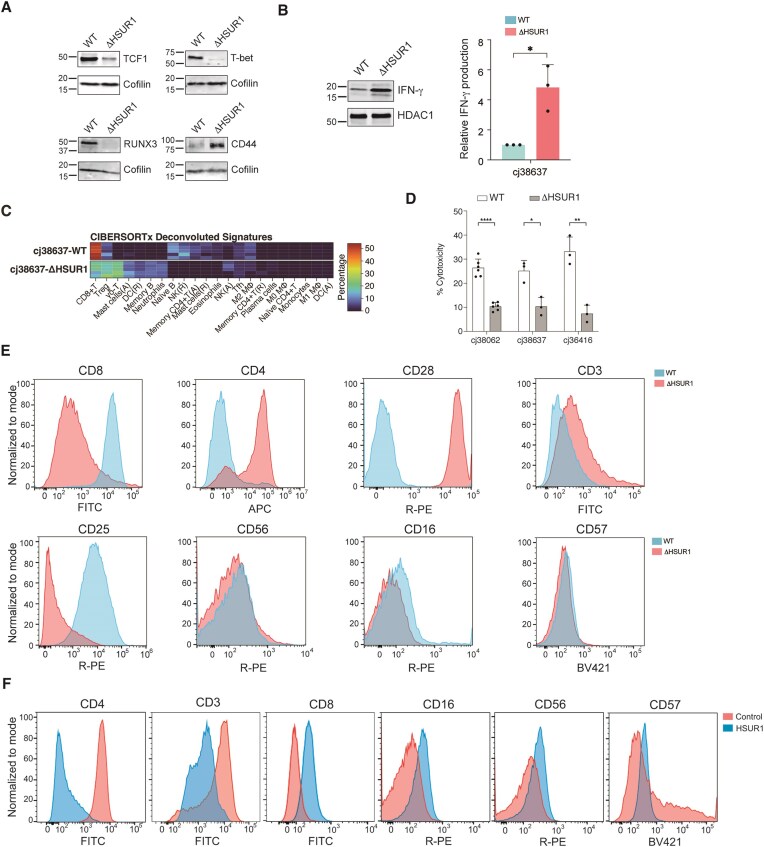
HSUR1 determines cell identity and function. (**A**) Western blot of representative HSUR1 target proteins in cj38637-WT and cj38637-ΔHSUR1 cells; cofilin serves as a loading control. (**B**) IFN-γ production in cj38637-WT and cj38637-ΔHSUR1 cells detected by western blot after PMA/ionomycin stimulation in the presence of brefeldin A and monensin; quantification of three replicates shown at right. HDAC1 provides a loading control. (**C**) Predicted composition of resting (R) and active (A) immune cell types inferred from gene expression using CIBERSORTx. T-reg, regulatory T cell; γδ-T, gamma-delta T cell; Tfh, T follicular helper cell; DC, dendritic cell; MΦ, macrophage; NK, natural killer cell. (**D**) NK-like cytotoxic activity of WT and ΔHSUR1 cells toward MOLT-4 target cells measured by CytoTox-Glo assay. (**E**) Flow cytometry analysis of cj38637-WT and cj38637-ΔHSUR1 cells for the indicated surface markers. (**F**) Same as in panel (E) for the indicated markers in HuT78 cells (Control) or same cells transduced with a lentiviral vector expressing HSUR1. *P < .05, **P < 0.01, ****P < .0001.

No statistical methods were used to predetermine sample size, nor were the experiments randomized or the investigators blinded to sample allocation during experiments and evaluation of experimental results. “Biological replicates” (*n*), indicated in figures and figure legends, refers to the number of independent experiments performed. The number of independent experiments was chosen to allow for statistical significance. Statistical analysis was performed using GraphPad Prism 10. For calculation of average free energy of binding (Δ*G*) of interactions between HSUR1 and HSUR1-binding sites located at the 3′UTRs of target transcripts (*n* = 313) compared with the mean Δ*G* of length-matched, random 3′UTR sequences (1000 random permutations per binding site), statistical significance was determined with a two-sided unpaired *t*-test with Welch’s correction for unequal variances. Uncorrected, two-sided *P*-values of biological replicates shown in Figs 5F (HeLa-H1 miR-142-3p versus HeLa-H1-AREm1 or AREm2 miR-142-3p), 6B, and 7D; Supplementary Figs S2C and S6A were obtained with multiple unpaired Student’s *t*-tests. Uncorrected, two-sided *P*-values of biological replicates shown in Figs. 3D, E, 5A, 6C and Supplementary Figs S3B, C, S5D, E, F, and G were obtained with multiple unpaired Student’s *t*-tests and compared to the controls set as 1.0. Statistical analysis of biological replicates shown in Fig. 6D was performed using a two-way analysis of variance (ANOVA, alpha = 0.05) that showed significant difference among the means (*P *< .0001) followed by an uncorrected Fisher’s LSD test for each comparison. Two-sided statistical analyses of biological replicates shown in Figs 2A, C, D, 3B and C, and in all panels of Fig. 4, as well as in Supplementary Fig. S4A, were performed with multiple Student’s *t-*tests corrected for multiple comparisons with the Holm-Šídák method (alpha = 0.05) and compared to the controls set as 1.0. The pairwise comparisons made between HSUR1, HSUR1 ARE mutants, and the control shown in Fig. 5C and Supplementary Fig. S5C, or between HSUR1 and HSUR1 mutants shown in Supplementary Fig. S5B, were performed using an ordinary one-way ANOVA test that showed significant difference among the means (*P *< .0001) followed by an uncorrected Fisher’s LSD test for each comparison. Uncorrected two-sided *P*-values for the pairwise comparisons of biological replicates in Fig. 5E were calculated with rank comparisons using the Mann–Whitney test. Two-tailed *P*-values for the production of IFN-γ by ΔHSUR1 cells shown in Fig. 7B and Supplementary Fig. S7B and E relative to the controls (marmoset cells infected with WT HVS) set as 1.0 were obtained with one-sample Student’s *t*-test for each cell line. Two-tailed *P*-values for the comparison of V5 IPs from HeLa-H1 cells in the presence of miR-142-3p relative to a theoretical mean for the control (HeLa-C transfected with miR-142-3p) set as 1.0 (Fig. 5F) were obtained by a one-sample Student’s *t*-test for each gene. Exact *P*-values for all statistical tests performed on the data presented in the manuscript are listed in Supplementary Table S6.

## Results

### HSUR1 binds and regulates host mRNAs in infected cells

Most known functions of Sm-class RNAs rely on base pairing with pre-mRNAs or mature mRNAs [7, 24]. Because HSUR1 interacts with both miRNAs and ARE-BPs that regulate mRNA stability and translation, we hypothesized that HSUR1 modulates gene expression by base-pairing with target mRNAs and recruiting these trans-acting regulators. To test whether HSUR1 engages mRNAs *in vivo*, we performed AMT-mediated RNA–RNA crosslinking in HVS-A11–transformed marmoset T cells (cj319-WT), which express all HSURs [6], followed by oligo-dT selection. HSUR1 was recovered in complexes containing polyadenylated (polyA⁺) RNA (Fig. 1B), indicating that it base pairs with mature mRNAs in infected cells. Similar to HSUR2, a related viral snRNA that recruits miRNAs to their targets and associates with actively translated mRNAs [7], HSUR1 co-sedimented with miRNAs and polysomes upon sucrose-gradient fractionation (Supplementary Fig. S1A), suggesting that HSUR1 also associates with translating mRNAs.

To define HSUR1–mRNA interactions transcriptome-wide, we used iRICC (individual-nucleotide resolution RNA–RNA interaction identification by crosslinking and capture) [8] in cj319-WT cells. iRICC identified 867 host transcripts that base pair with HSUR1 (Supplementary Table S1), ∼96% of which were protein-coding mRNAs (Fig. 1C), indicating that mRNA regulation is a primary function of HSUR1. We mapped 2303 discrete HSUR1 peaks corresponding to binding sites across these mRNAs (Supplementary Table S1). Most interactions localized to coding sequences (CDS, 61.7%), with additional sites positioned in 5′ UTRs or 5′ UTR/CDS boundaries (∼23%) and in 3′ UTRs or 3′ UTR/CDS boundaries (∼13%) (Fig. 1D). Approximately 75% of HSUR1-targeted mRNAs contained multiple binding sites, with many transcripts displaying several binding sites distributed along their entire length (Supplementary Table S1).

### HSUR1 represses target mRNAs through their 3′UTRs

HSUR1 binds miRNAs and ARE-BPs, which typically act through 3′ UTR elements to regulate mRNA stability. This suggested that HSUR1 may function as an adaptor that delivers miRNAs and ARE-BPs to the 3′ UTRs of target transcripts to modulate their expression. To examine how HSUR1 affects host gene expression, we generated three matched sets of marmoset T-cell lines transformed de novo with either WT HVS-A11 (cj36416-WT, cj38062-WT, cj38637-WT) or an HSUR1-deletion mutant (cj36416-ΔHSUR1, cj38062-ΔHSUR1, cj38637-ΔHSUR1) (Supplementary Fig. S2A), enabling direct comparison of cellular phenotypes in the presence or absence of HSUR1.

To test whether HSUR1 directly regulates target mRNAs by tethering miR-142-3p and ARE-BPs, we focused on transcripts bound via binding sites located exclusively within their 3′UTRs (Fig. 1E and Supplementary Fig. S11) to rule out the possibility that the effects observed upon expression of HSUR1 are due to other potential mechanisms employed by this viral snRNA when it binds to coding sequences or 5′ UTRs. In cj38637-WT cells, HSUR1 expression correlated with reduced abundance of this class of targets as measured by qRT-PCR (Fig. 2A), when compared to cj38637-ΔHSUR1 cells that do not express HSUR1 (Supplementary Fig. S2A). By contrast, no changes were observed for non-target control mRNAs that do not associate with HSUR1 according to iRICC and thus are not expected to be rapidly affected by transient expression or depletion of HSUR1. Lower abundance of HSUR1 target mRNAs in cj38637-WT cells correlated with lower protein levels when compared to cj38637-ΔHSUR1 cells (Fig. 2B and Supplementary Fig. S2B). Transient knockdown of HSUR1 with an antisense oligo nucleotide (ASO) (Supplementary Fig. S2C) in cj319-WT cells resulted in increased HSUR1 target mRNA abundance (Fig. 2C). Since identified HSUR1 binding sites are mostly conserved in human homolog genes (Supplementary Fig. S8), we transfected a plasmid expressing HSUR1 in a human cell line to test whether HSUR1 alone can repress target mRNA expression. Ectopic expression of HSUR1 in the human T-cell lymphoma line HuT78, which naturally expresses miR-142-3p, was sufficient to reduce target transcript abundance (Fig. 2D). Together, these data demonstrate that HSUR1 autonomously represses host mRNAs.

iRICC identified 176 HSUR1 peaks within 3′ UTRs and 121 peaks spanning CDS/3′ UTR boundaries corresponding to 313 potential binding sites for HSUR1 within these regions in 213 target transcripts (Supplementary Table S1), with most target transcripts harboring multiple sites. These interactions show on average a lower free energy of binding than that expected by chance (Supplementary Fig. S1C). Sequence analysis of HSUR1–mRNA interactions revealed that HSUR1 does not use a single seed sequence; instead, similar to HSUR2 [8], it base-pairs with target mRNAs through variable stretches of nucleotides within an extended domain between the ARE and the Sm-binding site (Supplementary Fig. S3A), forming distinct base-pairing configurations with individual binding sites (Supplementary Table S1). In luciferase reporter assays in which HSUR1 was transiently expressed in HuT78 cells that constitutively express luciferase reporter transcripts (Fig. 3A), HSUR1 did not repress transcripts lacking a 3′ UTR, indicating that HSUR1 cannot affect the transcription, translation, and/or stability of the firefly luciferase reporter transcript. In contrast, expression of HSUR1 resulted in lower expression of luciferase reporter genes carrying partial 3′ UTR sequences from HSUR1 targets containing multiple binding sites (Fig. 3B), recapitulating the effect observed on endogenous target mRNAs (Fig. 2C and D). Short 3′ UTR fragments containing single binding sites exhibited variable repression (Fig. 3C), suggesting that sequence context or additional 3′ UTR elements are important for HSUR1 activity.

We performed mutational analyses to confirm that the interactions between HSUR1 and target mRNAs are mediated by the arrangements of base pairs predicted by iRICC. Mutating target mRNA sequences involved in HSUR1–mRNA base-pairing abolished repression by WT HSUR1 (Fig. 3D and E, and Supplementary Fig. S3B and C). Conversely, expression of mutant versions of HSUR1 carrying compensatory mutations predicted to restore complementarity with the mutant 3’UTRs restored repression in every case (Fig. 3D and E, and Supplementary Fig. S3B and C). These results indicate that base pairing with the target transcript is required for HSUR1-mediated repression through 3′ UTRs and confirm that HSUR1 directly repress target mRNAs through specific base pairing identified by iRICC.

### HSUR1-mediated mRNA repression requires miR-142-3p and cooperation with ARE-binding proteins

HSUR1 lowers the abundance of a subset of target mRNAs by binding to their 3′UTRs. To determine whether HSUR1-associated miRNAs and ARE-BPs contribute to target mRNA repression, we examined HSUR1 function in HeLa-H1 cells, which express HSUR1 but lack miR-142-3p [12] and where we could detect the expression of all analyzed HSUR1 target mRNAs and control mRNAs by qRT-PCR. In the absence of miR-142-3p, HSUR1 alone did not affect target mRNA abundance (Fig. 4A). Transient expression of miR-142-3p in control HeLa cells that do not express HSUR1 (HeLa-C) had no effect on the abundance of HSUR1 target mRNAs, indicating that these are not regulated by miR-142-3p. Conversely, transient expression of miR-142-3p in HeLa-H1 cells induced robust repression of HSUR1 targets, demonstrating that miR-142-3p is essential for HSUR1-mediated mRNA repression. A 5′-end mutant of HSUR1 defective for miR-142-3p binding failed to repress target mRNAs, whereas a compensatory miR-142-3p mutant restored repression (Fig. 4B and Supplementary Fig. S4A), confirming that direct HSUR1–miR-142-3p interaction is required for target mRNA repression.

Binding of miR-27 to HSUR1 triggers its degradation by TDMD [6, 10] and a majority of HSUR1 binding sites in target 3′UTRs (225 out of 313) are predicted to overlap with the miR-27 binding site present in HSUR1 (Supplementary Table S1). This suggests that HSUR1 does not utilize miR-27 to repress target mRNAs. We used a LNA inhibitor to miR-27, which efficiently inhibits this miRNA in HeLa-H1 cells [12], to test this hypothesis. Transient inhibition of miR-27 did not alter target mRNA abundance, either in the absence or presence of miR-142-3p (Fig. 4C), indicating that miR-27 is dispensable for HSUR1-mediated mRNA repression.

To determine whether ARE-BPs contribute to HSUR1 function, we surveyed expression of several ARE-BPs reported to regulate mRNA stability [25] in the cell lines used in this study. KHSRP, HuR, hnRNP D, and the TTP-related Zinc Finger Protein 36 Like 1 (ZFP36L1) were detectable in all cell lines tested, whereas TTP and ZFP36L2 were not, even though the antibodies employed could detect these proteins when they were transiently expressed in HeLa cells (Supplementary Fig. S4B and C). To test whether ARE-BPs could contribute to HSUR1-mediated mRNA repression, we decided to transiently overexpress ZFP36L2, which is not detected in any of the cells, to be able to attribute changes in HSUR1-mediated repression to the ectopically expressed protein and not to endogenously expressed ARE-BPs. Using luciferase reporters constitutively expressed in HuT78 cells (which express endogenous miR-142-3p) carrying partial 3′UTRs of HSUR1 target mRNAs *JARID2, ADAR, HNRNPA2B1*, and *PPFIA1* (Fig. 3) that lack canonical AREs, we found that ZFP36L2 alone had no effect on reporter levels, indicating that this protein does not regulate these reporter transcripts (Fig. 4D). Transient expression of HSUR1 alone resulted in repression, whereas co-expression of ZFP36L2 with HSUR1 further enhanced repression (Fig. 4D). These results indicate that ARE-BPs can contribute to HSUR1-mediated mRNA repression, and that binding to HSUR1 allows ARE-BPs to repress mRNA transcripts that do not carry AREs.

Binding of miR-142-3p is required for binding of miR-27 to HSUR1 and binding of miR-16 to HSUR2 [12]. Thus, we hypothesized that miR-142-3p might also be required for the use of ARE-BPs in HSUR1-mediated mRNA repression. We tested this hypothesis by expressing ZFP36L2 together with either Control miRNA or miR-142-3p, in HeLa-H1 cells (Fig. 4E), which do not endogenously express miR-142-3p or ZFP36L2. Expression of ZFP36L2 with Control miRNA did not enhance repression of HSUR1 target mRNAs, suggesting that HSUR1 cannot engage ARE-BPs for target mRNA repression in the absence of miR-142-3p. In contrast, co-transfection of miR-142-3p with a control plasmid (mCherry) resulted in repression of target mRNAs, recapitulating the results obtained in Fig. 4A. Co-expression of miR-142-3p and ZFP36L2 showed a trend of enhanced repression compared to cells transfected with miR-142-3p and control plasmid for most, but not all, HSUR1 target mRNAs. Collectively, these results suggest a two-step mechanism for HSUR1-mediated mRNA repression: binding of miR-142-3p to HSUR1 is essential for downregulation of target transcripts, and this interaction enables HSUR1 to recruit ARE-BPs to 3′UTRs that are otherwise insensitive to these proteins. This mechanism allows HSUR1 to selectively repress target mRNAs independently of miR-27 and canonical ARE elements.

### Dual role for the ARE in HSUR1 stability and target mRNA repression

HSUR1 contains a canonical ARE that triggers its own degradation in cell types lacking miR-142-3p [16]. Paradoxically, HSUR1 cannot engage ARE-BPs to repress target mRNAs in the absence of this miRNA (Fig. 4E). We hypothesized that HSUR1 partitions into at least two populations: one that binds miR-142-3p and ARE-BPs to mediate target mRNA repression, and a second population that is regulated by ARE-BPs independently of miR-142-3p. Supporting this model, transient siRNA-mediated knockdown of KHSRP or hnRNP D, but not HuR, in HeLa-H1 cells lacking miR-142-3p increased HSUR1 abundance (Fig. 5A and Supplementary Fig. S5A and B), indicating an ARE-BP–regulated pool of HSUR1 that is incompetent for target mRNA repression.

We next investigated the role of HSUR1’s ARE in HSUR1-mediated mRNA repression. HSUR1’s ARE comprises three consecutive AUUUA pentamers (Fig. 1A). To dissect their contribution to mRNA repression, we generated three HSUR1 mutants, each disrupting a single pentamer through a U→G substitution (Fig. 5B). All mutants displayed elevated expression in HeLa cells (Supplementary Fig. S5B), consistent with impaired ARE-BP binding. In luciferase reporter assays with ZFP36L2 co-expression in HuT78 cells that naturally express miR-142-3p, WT HSUR1 maximally repressed the PPFIA1 (Fig. 5C) and ADAR (Supplementary Fig. S5C) reporters, whereas disruption of pentamers 1 or 2 led to partial repression when compared to cells transfected with a Control plasmid without HSUR1 (Fig. 5C and Supplementary Fig. S5C), even though the two mutant constructs are expressed at higher levels (Supplementary Fig. S5B). Disruption of the third AUUUA pentamer completely abolished repression on the PPFIA1 and ADAR reporters, phenocopying the HSUR1 mutant that is unable to bind miR-142-3p (Figs 4B and 5C, and Supplementary Fig. S5C). Co-immunoprecipitation with antibodies against Sm proteins [6, 12] on extracts from HeLa cells constitutively expressing either WT or mutant HSUR1s and transiently transfected with miR-142-3p demonstrated that pentamer 1 and pentamer 2 mutants retained miR-142-3p binding, whereas pentamer 3 mutant showed markedly reduced miR-142-3p association despite similar expression levels (Fig. 5D). These results indicate that the third ARE pentamer is essential for miR-142-3p binding. They also indicate that miR-142-3p and ARE-BP do not cooperatively bind to HSUR1 and suggest a mechanism whereby miR-142-3p binding licenses ARE-BP recruitment: maximal repression requires both miR-142-3p and intact ARE pentamers, intermediate repression occurs when pentamers 1 or 2 are disrupted and HSUR1 can bind miR-142-3p but not ARE-BPs, while loss of pentamer 3 prevents miRNA binding and target repression entirely.

To examine whether HSUR1 recruits ARE-BPs to mRNA targets, we performed immunoprecipitations in cj38637-WT and cj38637-ΔHSUR1 cells using antibodies to ARE-BPs. HSUR1 target transcripts, but not control mRNAs, were selectively enriched in KHSRP (Fig. 5E), HuR (Supplementary Fig. S5D), and hnRNP D (Supplementary Fig. S5E) immunoprecipitates from cj38637-WT cells relative to cj38637-ΔHSUR1 cells. In contrast, no differential enrichment of HSUR1 target mRNAs was observed when comparing immunoprecipitates with normal rabbit control serum on extracts prepared from the same cells (Supplementary Fig. S5F). Because commercial ZFP36L1 antibodies were inefficient for precipitation, we transiently expressed GFP-ZFP36L1 in control HeLa (HeLa-C) and HeLa-H1 cells together with miR-142-3p and performed immunoprecipitations with antibodies against GFP on extracts prepared from these cells (Supplementary Fig. S5G). HSUR1 target mRNAs were enriched in GFP-ZFP36L1 complexes precipitated with GFP antibodies from HeLa-H1 but not from HeLa-C cells (Supplementary Fig. S5G), indicating that HSUR1 promotes recruitment of multiple ARE-BPs to its target mRNAs.

To test the mechanistic requirements for miR-142-3p and the ARE in HSUR1-mediated ARE-BP recruitment to target mRNAs, we co-transfected HeLa-C, HeLa-H1, and HeLa cells constitutively expressing a HSUR1 ARE-mutant (HeLa-H1AREm1 or HeLa-H1AREm2) with V5-tagged ZFP36L2 and either Control or miR-142-3p miRNA and immunoprecipitated extracts from these cells with antibodies against the V5 epitope. HSUR1 target mRNAs were enriched in V5-ZFP36L2 immunoprecipitates only in cells expressing WT HSUR1 and miR-142-3p (Fig. 5F), whereas ARE mutants failed to recruit ARE-BPs even in the presence of miR-142-3p. Collectively, these data define a mechanism in which HSUR1-mediated repression is governed by interactions between its ARE and miR-142-3p. Binding of miR-142-3p licenses HSUR1 to bind and recruit ARE-BPs to target mRNAs. Maximal repression requires engagement of both miR-142-3p and intact ARE pentamers; partial repression occurs when pentamers 1 or 2 are disrupted and HSUR1 cannot recruit ARE-BPs but can still engage miR-142-3p, whereas loss of pentamer 3 prevents miR-142-3p binding and target repression completely.

### HSUR1 broadly reprograms host gene expression and promotes cell survival

GO and KEGG analyses of HSUR1-associated mRNAs revealed enrichment for cell cycle, immune response, and apoptosis pathways (Fig. 6A and Supplementary Table S2), all processes crucial for maintenance of HVS latency [26]. Notably, a disproportionately large fraction of HSUR1 targets encode regulators of gene expression, including core spliceosomal components, SR proteins, hnRNPs, chromatin modifiers, and transcriptional and translational regulators, suggesting that HSUR1 sits upstream of multiple layers of post-transcriptional and epigenetic control. Additional targets involved in T-cell identity and differentiation (Supplementary Tables S1 and S2) suggest that HSUR1 may influence the identity of latently infected cells.

Because HSUR1 directly interacts with several mRNAs encoding apoptosis regulators, including caspase-6 and TGFβ (Supplementary Table S1), we tested whether HSUR1 expression might translate into functional control of cell survival. The three independent sets of marmoset T cells transformed in parallel with either WT HVS-A11 or ΔHSUR1 virus showed a consistent phenotype: HSUR1 expression increased cell viability and decreased apoptosis and necrosis as revealed by staining with propidium iodide and annexin V (Fig. 6B and Supplementary Fig. S6A). HSUR1-deficient cells exhibited stronger activation of caspase-3/7 following Fas ligand stimulation (Fig. 6C), consistent with selective de-repression of pro-apoptotic HSUR1 targets in the absence of this viral snRNA. The consistency observed among the three sets of newly transformed cell lines strongly suggests that these phenotypic differences are due to the absence of HSUR1 and not to stochastic events that occurred during the transformation process. Furthermore, ectopic expression of HSUR1 in human HuT78 T cells recapitulated this phenotype (Fig. 6D), indicating that HSUR1 is sufficient to suppress apoptotic signaling.

To define the global regulatory impact of HSUR1, we performed RNA-seq on cj38637-WT and cj38637-ΔHSUR1 cells in five biological replicates. HSUR1 altered the abundance of ∼24% of expressed mRNAs by ≥2-fold and ∼37% by ≥ 1.5-fold in virally transformed cells (Fig. 6E and Supplementary Table S3), underscoring its extensive influence on host gene expression. GO analysis of differentially expressed genes (Supplementary Fig. S9 and Supplementary Table S8) shows enrichment in pathways also related to apoptosis and cell death, immune function and NK-cell mediated cytotoxicity. These pathways were also enriched in the GO and KEGG analyses of direct targets of HSUR1 (Fig. 6A and Supplementary Table S2), indicating that indirect regulation of gene expression by HSUR1 might reinforce phenotypic effects on these pathways. Notably, only 187 of the 867 (∼21%) direct HSUR1 targets showed significant abundance changes, indicating that most HSUR1–mRNA interactions regulate gene expression through mechanisms that do not strongly alter steady-state mRNA levels. Given the established role of HSUR1 in promoting miR-27 degradation [6, 27], we examined miR-27 targets identified by high-throughput sequencing of RNA after crosslinking and immunoprecipitation (HITS-CLIP) in cj319-WT cells [28]. Only 12 of these transcripts—including SEMA7A—were derepressed in HSUR1-expressing cells (Supplementary Table S3), suggesting that miR-27 degradation contributes modestly to the overall transcriptome reprogramming induced by HSUR1. These observations indicate that the broad gene expression changes driven by HSUR1 are largely pleiotropic and likely mediated through its effects on mRNAs encoding key gene expression regulators.

Because HSUR1 targets include numerous splicing regulators—such as SR proteins, hnRNPs, and core spliceosomal components (Supplementary Tables S1 and S2)—we next examined its impact on alternative splicing in HVS-infected cells. Transcriptome-wide analysis of cj38637-WT and cj38637-ΔHSUR1 revealed 840 differential splicing events affecting 706 genes, including intron retention, alternative 3′/5′ splice sites selection, and cassette exon inclusion (Fig. 6F and G; Supplementary Table S4). Only 59 HSUR1-bound mRNAs displayed altered splicing, indicating that the vast majority of splicing changes arose indirectly, likely due to HSUR1-dependent modulation of splicing factor expression. HSUR1 depletion led to pronounced splicing defects, including increased intron retention in 107 of 120 regulated introns (Fig. 6F) and preferential usage of distal splice sites at both 3′ and 5′ junctions. Of the 490 differentially spliced cassette exons, 269 (∼54%) displayed higher inclusion in HSUR1-expressing cells (Fig. 6G). Remarkably, nearly one-third (29.7%) of these exons were not previously annotated as alternatively spliced in the marmoset genome and exhibited higher percent spliced-in (PSI) values than annotated alternative exons (Supplementary Fig. S6B), suggesting that HSUR1 activates cryptic or low-frequency splicing events. In contrast, most differential intron retention events displayed high retention levels in both cj38637-WT and cj38637-ΔHSUR1 cells (Supplementary Fig. S6C). Most regulated coding exons preserved reading frame (242 of 358, 67%) and showed strong conservation with human orthologs, consistent with potential functional relevance. Importantly, only 6% of differentially expressed genes also exhibited differential splicing, indicating that most HSUR1-induced changes in mRNA abundance occur independently of splicing alterations. These findings support a model in which HSUR1 rewires host splicing programs indirectly by modulating the expression of multiple splicing regulators, leading to widespread yet mechanistically diverse splicing outcomes.

### HSUR1 determines cell identity and function

Many HSUR1-bound transcripts encode lineage-defining transcription factors (TFs) that orchestrate T-cell development and effector specification [29], including TCF1, BCL11B, T-bet (*TBX21*), Ikaros family members, and RUNX3 (Supplementary Table S1)—suggesting that HSUR1 directly engages the gene-regulatory circuits that determine T-cell identity. Consistent with this model, HSUR1-expressing cj38637-WT cells displayed higher abundance of TCF1, T-bet, and RUNX3 mRNAs compared to cj38637-ΔHSUR1 cells (Supplementary Table S3), and these changes were mirrored at the protein level in HSUR1-expressing cells (Fig. 7A). The upregulation observed on these target mRNAs, which carry HSUR1 binding sites in their coding sequences, is the opposite to that observed on mRNAs that carry HSUR1 binding sites in their 3′UTRs (Fig. 2). Equivalent effects observed for the other two independently transformed cell sets (Supplementary Fig. S7A and D) indicate that HSUR1 consistently reinforces expression of multiple lineage-defining TFs.

We next tested whether HSUR1 similarly regulates other direct targets that influence cellular phenotypes. Although CD44 mRNA, which is bound by HSUR1 at its 5′UTR, multiple times in its coding region, and its 3′UTR (Supplementary Table S1), shows no change in abundance upon expression of HSUR1 (Supplementary Table S3), CD44 protein levels decreased in HSUR1-expressing cells (Fig. 7A and Supplementary Fig. S7A and D), further underscoring the complexity of HSUR1 regulation when binding to different regions of an mRNA.

We next wanted to investigate the effect of HSUR1 on cytokine production. Like RUNX 3 mRNA, IFN-γ mRNA carries one HSUR1 binding site in its coding sequence (Supplementary Table S1 and Supplementary Fig. S10). HSUR1 expression consistently correlated with reduced IFN-γ production after phorbol 12-myristate 13-acetate (PMA)/ionomycin stimulation (Fig. 7B and Supplementary Fig. S7B and E), showing the opposite effect to that observed on RUNX3 mRNA and indicating that HSUR1 modulates effector cytokine output in addition to lineage-specifying TF expression. Since HSUR1 base pairs with the mRNAs encoding these proteins, the effect observed over the expression of these factors could be attributed, at least partially, to direct regulation of HSUR1 over these target mRNAs. This would suggest that HSUR1 can act through different mechanisms that could be specific to a region or identity of the mRNA. However, it is also possible that the effects observed are the result of direct regulation by HSUR1 combined with indirect effects of HSUR1-mediated regulation of other gene expression factors. Altogether, these data highlight HSUR1 as a multifunctional post-transcriptional regulator that tunes both the transcriptional architecture and functional effector programs of virally infected cells.

HVS-transformed cells have been characterized as predominantly cytotoxic CD8^+^ T cells that show little to no expression of the CD4 marker and that express the NK cell marker CD56 [30, 31]. We used CIBERSORTx [32], a machine learning method that uses gene expression data to infer the abundance of member cell types in a mixed cell population, to deconvolute the RNA-seq data from cj38637-WT and cj38637-ΔHSUR1 cells. This analysis revealed that marmoset cells transformed with WT HVS exhibited expression signatures characteristic of cytotoxic CD8^+^ T cells (Fig. 7C). Loss of HSUR1, however, shifted expression profiles toward regulatory T (Treg) and γδ T-cell states, suggesting that HSUR1 is required to maintain a CD8⁺ T-cell-like identity.

HVS-transformed cells present NK-like cytotoxic activity and can spontaneously kill MOLT-4 cells [30, 31]. GO analyses identified HSUR1 targets encoding mediators of NK- and CD8-cell cytotoxicity [33] (Fig. 6A), including Granzyme B and Cathepsin W (Supplementary Tables S1 and S2), both elevated in HSUR1-expressing cells (Supplementary Table S3). Accordingly, HSUR1-expressing cells exhibited higher killing of MOLT-4 targets than ΔHSUR1 cells (Fig. 7D), indicating that HSUR1 enhances the cytotoxic capacity of transformed T cells. Altogether, these results suggest that HSUR1 couples post-transcriptional regulation of lineage-defining TFs with modulation of effector molecules to enforce a cytotoxic identity. Flow-cytometric analyses of newly transformed cells further supported this model. HSUR1 loss resulted in reduced CD8, and increased CD4 expression (Fig. 7E and Supplementary Fig. S7C and F), reinforcing the notion that HSUR1 drives a CD8⁺ cytotoxic phenotype while inhibiting differentiation into CD4⁺ Treg-like states. HSUR1 also upregulated expression of the interleukin-2 receptor (CD25), a direct HSUR1 target involved in T-cell activation, and suppressed CD28 and CD3, consistent with broad remodeling of T-cell activation and signaling pathways. Among NK markers, CD16 was selectively increased in HSUR1-expressing cells, while CD56 and CD57 remained unchanged. Importantly, ectopic HSUR1 expression in HuT78 cells recapitulated these effects, decreasing CD3 and CD4 expression while increasing expression of CD8 and NK-associated markers (CD16, CD56, CD57), suggesting that HSUR1 can autonomously reprogram immune-cell identity independent of viral transformation context. Altogether, these results identify HSUR1 as a regulatory hub that integrates post-transcriptional control of lineage-specifying TFs, effector molecules, and cytokine programs to enforce a cytotoxic CD8⁺-like state in infected cells.

## Discussion

Our findings establish HSUR1 as a master regulator of host gene expression in HVS-transformed T cells. HSUR1 acts through an unexpectedly diverse set of mechanisms, directly base-pairing with cellular mRNAs across untranslated regions and coding sequences to modulate their stability and possibly translation and other aspects of RNA metabolism. Most of the transcripts that interact with HSUR1 correspond to protein-coding mRNAs (Fig. 1C); it is possible, however, that interactions with non-polyadenylated transcripts may have been missed since iRICC was performed on an RNA fraction enriched for polyadenylated RNAs. Analysis of the distribution of binding sites for HSUR1 on target mRNAs revealed that HSUR1 binds to all mRNA regions, with many target mRNAs presenting multiple binding sites, and with most binding sites located in the CDS (Fig. 1D). This binding pattern is different from the one revealed by iRICC for the related viral snRNA HSUR2, which binds most target mRNAs just once, mostly on their 3′UTRs [8]. The different binding patterns of these two related but distinct viral snRNAs highlight the specificity and reliability of iRICC as a method that confidently identifies RNA–RNA interactions *in* vivo. Since HSUR1 can bind all regions of target mRNAs, we speculate that this viral snRNA regulates targets through multiple mechanisms. Like HSUR2 and unlike miRNAs, HSUR1 does not utilize a fixed seed region to interact with target mRNAs but instead forms target-specific base-pairing arrangements using distinct residues and conformations (Fig. 3 and Supplementary Fig. S3, and Supplementary Table S1). Also, similarly to HSUR2 [8], there is no clear correlation between binding affinity and the extent of repression exerted by HSUR1 on target mRNAs (Fig. 3, Supplementary Fig. S3, and Supplementary Table S1). Although this flexible pairing capacity theoretically permits complementarity to a large fraction of the transcriptome, only a subset of potential targets is engaged *in vivo*, suggesting that base-pairing is necessary but not sufficient for target recognition. Additional features in the mRNA or auxiliary protein cofactors likely guide HSUR1 to specific transcripts, similarly to cellular Sm-class RNAs that participate in splicing [34–39].

Our mapping of HSUR1 binding sites revealed widespread interactions within 5′UTRs, 3′UTRs, and especially coding sequences (Fig. 1D and Supplementary Fig. S1B). This distribution correlates with diverse regulatory outcomes. Binding to 3′UTRs triggers a defined mechanism in which HSUR1 recruits miR-142-3p and ARE-BPs to repress target mRNAs (Figs 2–5 and Supplementary Figs S2–S5). Expression of HSUR1 results in lower levels of target mRNAs in a miR-142-3p-dependent fashion, while ARE-BPs can enhance this effect (Figs 2 and 4). The simplest model to explain these observations is that HSUR1-mediated recruitment of miR-142-3p and ARE-BPs to target mRNAs directly promotes their degradation. In support of this model, both Ago proteins and ARE-BPs can elicit transcript degradation when artificially tethered to mRNAs [40, 41]. However, since HSUR1 regulates the expression of transcription factors as well as other gene expression factors, it is possible that the effects observed on mRNA and protein abundance of the HSUR1 targets analyzed in this study are a result of a combination of direct and/or indirect regulatory mechanisms exerted by HSUR1. Similarly to HSUR2-mediated mRNA repression [7, 12] and HSUR1-directed miR-27 degradation [12], HSUR1-mediated repression through 3′UTRs is dependent on a miRNA binding to the 5′ end of the snRNA, further underscoring the central role of miR-142-3p in HSUR snRNA function. Because miR-142-3p is restricted to hematopoietic lineages [42, 43], its requirement likely ensures that HSUR1 activity—and therefore HVS latency—remains confined to immune cells. We speculate that miR-142-3p binding induces conformational changes that expose HSUR1’s ARE and miR-27 binding site, consistent with *in vivo* chemical probing revealing that HSUR1 is structurally highly dynamic [27].

HSUR1 interactions in 5′UTRs, a region often involved in translational control [44], may influence translation initiation, whereas binding within coding sequences likely engages distinct mechanisms. Indeed, the divergent effects on RUNX3 and IFN-γ mRNAs (Fig. 7A and Supplementary Fig. S7A and D), each containing a single CDS binding site, illustrate that HSUR1 can either promote or repress expression depending on target context. Given that most targets harbor multiple binding sites, individual transcripts may be subject to combinatorial layers of HSUR1-mediated regulation as suggested above. HSUR1-directed recruitment of miRNAs and ARE-BPs to coding regions may also contribute to repression [45–47] or influence other processes such as mRNA export [48]. Since HSUR1 can recruit all tested ARE-BPs to target mRNAs (Fig. 5E and Supplementary Fig. S5D, E, and G), it is possible that they can redundantly contribute to HSUR1-mediated mRNA repression when HSUR1 binds target mRNAs in 3′UTRs. Alternatively, the heterogeneity of base-pairing interactions could allow HSUR to preferentially recruit different ARE-BPs depending on the HSUR1–mRNA base-pairing geometry, with posttranslational modifications of ARE-BPs [49] adding another regulatory dimension.

Transcriptome-wide analyses revealed HSUR1’s pervasive impact on gene expression. HSUR1 alters mRNA abundance for nearly one quarter of the transcriptome (Supplementary Table S3), yet the regulatory reach of HSUR1 likely extends further as exemplified by CD44, for which protein levels change without affecting mRNA abundance (Fig. 7A). GO and KEGG pathway analyses of direct targets of HSUR1 predicted roles in several pathways including RNA processing, transcriptional regulation, mRNA translation, chromatin regulation, T-cell differentiation, NK-cell cytotoxicity, and apoptosis (Fig. 6A and Supplementary Table S2). HSUR1 could therefore indirectly affect mRNA abundance by perturbing transcription and/or mRNA stability through direct regulation of mRNAs encoding transcription factors, chromatin factors, or RNA-binding proteins. Furthermore, GO analyses of differently expressed genes (Supplementary Fig. S9 and Supplementary Table S8) indicated that HSUR1 could modulate processes such as apoptosis and cell death, immune cell function, and NK-cell mediated cytotoxicity. Consistent with these predictions, HSUR1 inhibits apoptosis, modulates pre-mRNA splicing, and promotes NK-like cytotoxic activity. The influence of HSUR1 on cellular phenotypes is likely broader than currently appreciated, as suggested by the enrichment of multiple functional categories revealed through GO and pathway analyses that have yet to be experimentally tested.

Perhaps the most striking consequence of HSUR1 function is its capacity to define the identity and cytotoxicity of infected cells. HVS-transformed marmoset lymphocytes have been classified as cytotoxic CD8^+^ T cells expressing NK markers such as CD56 [30, 31], leading to the assumption that HVS establishes latency in this specialized subset of immune cells [50]. Our findings instead suggest that HSUR1 actively molds this phenotype by modulating master transcription factors, surface markers, and effector cytokines—including IFN-γ (Fig. 3, Supplementary Fig. S3, and Supplementary Table S1). HVS could transform progenitor lymphoid cells [51] and drive their differentiation toward CD8^+^, NK-like cells. Another possibility is that HVS transforms differentiated immune cells and drive them toward this phenotype. In support of this second possibility, HSUR1 can drive this change in human T-cell lymphoma line HuT78, as evidenced by change in expression of cell surface markers (Fig. 7F). By driving an apoptosis-resistant, NK-like cytotoxic program, HSUR1 may enable infected cells to eliminate immune cells they encounter, facilitating immune evasion and long-term viral persistence. This possibility raises the intriguing hypothesis that viral ncRNAs can sculpt immune cell identity to secure a favorable niche for latency.

In summary, HSUR1 functions as a viral master regulator that rewires host gene expression through integrated post-transcriptional mechanisms. By base-pairing with hundreds of cellular transcripts encoding factors with important roles in different aspects of gene expression, HSUR1 exerts multilayered control over the cellular gene expression program. Through coordinated regulation of transcription factors, cytotoxic effectors, and apoptosis pathways, HSUR1 converts infected T cells into NK-like, apoptosis-resistant effectors that promote viral persistence. These findings illustrate how a single viral RNA can orchestrate complex gene regulatory networks and suggest that similar strategies may be employed by cellular ncRNAs to sculpt immune and developmental programs.

## Declaration of generative AI and AI-assisted technologies in the writing process

During the preparation of this work the authors used ChatGPT-5 to improve readability of the manuscript. After using this tool/service, the authors reviewed and edited the content as needed and take full responsibility for the content of the published article.

## Supplementary Material

gkag472_Supplemental_Files

## Data Availability

All data are available in the main text or supplementary materials, except for the RNA-seq and iRICC-seq experiments. The NGS (next generation sequencing) data are available publicly as of the date of publication. The data have been deposited in the Gene Expression Omnibus under the accession number GSE291067. Further data that support the findings of this study are available from the corresponding author upon reasonable request.
