# Supplementary material for: A viral noncoding RNA is a master regulator of gene expression that defines host cell identity and function
Source: Nucleic Acids Res. 2026 May 11;54(9):gkag472. doi: 10.1093/nar/gkag472 (PMC13158664; doi:10.1093/nar/gkag472)
Supplement: gkag472_Supplemental_Files [file gkag472_supplemental_files.zip › Captions of Supplementary Tables.docx]

**Captions of Supplementary Tables**

**Supplementary Table 1**

This file contains a list of HSUR1 target and binding sites identified by iRICC and sequences involved in interactions between HSUR1 and binding sites in 3′UTRs.

**Supplementary Table 2**

This file contains all results from Gene Ontology and Kegg Pathway analyzes of direct targets of HSUR1.

**Supplementary Table 3**

This file contains gene expression data based on RNA-seq on cj38637-WT and cj38637-ΔHSUR1 cells.

**Supplementary Table 4**

This file describes results from the analysis of splicing in cj38637-WT and cj38637-ΔHSUR1 cells.

**Supplementary Table 5**

This file contains the sequences of oligonucleotides used in cloning.

**Supplementary Table 6**

This file contains the *P*-values for statistical tests in Figures.

**Supplementary Table 7**

This file contains a list of antibodies used in this study.

**Supplementary Table 8**

This file contains all results from Gene Ontology analysis of differentially expressed genes in cj38637-WT and cj38637-ΔHSUR1 cells.
