# Supplementary material for: A viral noncoding RNA is a master regulator of gene expression that defines host cell identity and function
Source: Nucleic Acids Res. 2026 May 11;54(9):gkag472. doi: 10.1093/nar/gkag472 (PMC13158664; doi:10.1093/nar/gkag472)
Supplement: gkag472_Supplemental_Files [file gkag472_supplemental_files.zip › Supplementary Table 7.docx]

TABLE S7

| ANTIBODY | SOURCE | IDENTIFIER | DILUTION | APPLICATION |
| --- | --- | --- | --- | --- |
| ADAR1 (D7E2M) rabbit monoclonal antibody | Cell Signaling Technology | Cat#14175 | 1:1,000 | Western blotting |
| HNRNPA2B1 Lot 00099098 rabbit polyclonal antibody | Proteintech | Cat#14813-1-AP | 1:2,500 | Western blotting |
| PIP4K2B rabbit polyclonal antibody | Cell Signaling Technology | Cat#9694 | 1:1,000 | Western blotting |
| UBA2 (D15C11) Lot 1 rabbit monoclonal antibody | Cell Signaling Technology | Cat#8688 | 1:1,000 | Western blotting |
| JARID2 (D6MX9) Lot 3 rabbit monoclonal antibody | Cell Signaling Technology | Cat#13594 | 1:500 | Western blotting |
| GFP Lot 00143536 rabbit polyclonal antibody | Proteintech | Cat#50430-2-AP | 5 µg per 50 µl of protein A beads per sample | IP |
| Tristetraprolin (D1I3T) Lot 1 rabbit monoclonal antibody | Cell Signaling Technology | Cat#71632 | 1:1,000 | Western blotting |
| ZFP36L1 (E6L6S) Lot 1 rabbit monoclonal antibody | Cell Signaling Technology | Cat#30894 | 1:1,000 | Western blotting |
| ZFP36L2 (E4M9Z) Lot 1 rabbit monoclonal antibody | Cell Signaling Technology | Cat#85891 | 1:1,000 | Western blotting |
| HuR Lot 00100116 rabbit polyclonal antibody | Proteintech | Cat#11910-1-AP | 1:1,000  4.5 µg per 100 µl of protein A beads | Western blotting IP |
| HNRNP D (2G9E6) mouse monoclonal antibody | Proteintech | Cat#68236-1-Ig | 1:5,000 | Western blotting |
| HNRNP D Lot 00020473 rabbit polyclonal antibody | Proteintech | Cat#12770-1-AP | 1:5,000  20 µg per 100 µl of protein A beads | Western blotting  IP |
| KHSRP (A7-B0) Lot ZH4429303A mouse monoclonal antibody (IgM) | Invitrogen | Cat#MA5-34921 | 1:1,000 | Western blotting |
| KHSRP (E2E2U) Lot 1 rabbit monoclonal antibody | Cell Signaling Technology | Cat#13398 | 2 µg per 100 µl of protein A beads | IP |
| V5-tag (SV5-Pk2) mouse monoclonal antibody | Bio-Rad | Cat#MCA2892 | 5 µg per 50 µl of protein A beads per sample | IP |
| Cofilin (D3F9) XP® rabbit monoclonal antibody | Cell Signaling Technology | Cat#5175 | 1:10,000 | Western blotting |
| IR-Dye® 680RD goat anti-rabbit IgG | LI-COR Biosciences | Cat#926-68071 | 1:20,000 | Western blotting |
| IRDye® 680RD donkey anti-mouse IgG | LI-COR Biosciences | Cat#926-68072 | 1:20,000 | Western blotting |
| IRDye® 680RD goat anti-mouse IgM | LI-COR Biosciences | Cat#925-68180 | 1:20,000 | Western blotting |
| CD28 (REA612) R-PE conjugated human IgG1 monoclonal antibody | Miltenyi Biotec | Cat# 130-124-002 | 1:50 | Flow cytometry |
| CD4 (S3.5) APC conjugated mouse IgG2a monoclonal antibody | Thermo Fisher Scientific | Cat# MHCD0405 | 1:20 | Flow cytometry |
| CD4 (S3.5) FITC  Conjugated mouse IgG2a monoclonal antibody | Thermo Fisher Scientific | Cat#MHCD0401 | 1:20 | Flow cytometry |
| CD3 (7D6) FITC conjugated mouse IgG2a monoclonal antibody | Thermo Fisher Scientific | Cat# MHCD0301 | 1:20 | Flow cytometry |
| CD8a (HIT8a) FITC conjugated mouse IgG1 monoclonal antibody | BioLegend | Cat# 300906 | 1:20 | Flow cytometry |
| CD16 (eBioCB16) R-PE conjugated mouse IgG1 monoclonal antibody | Thermo Fisher Scientific | Cat# 12-0168-42 | 1:20 | Flow cytometry |
| CD56 (39D5) R-PE conjugated mouse IgG1 monoclonal antibody | BioLegend | Cat# 355504 | 1:20 | Flow cytometry |
| CD57 (NK-1) BV421 conjugated mouse IgM | BD Biosciences | Cat# 563896 | 1:20 | Flow cytometry |
| CD25/IL2RA rabbit polyclonal antibody | Proteintech | Cat# 30449-1-AP | 1:100 | Flow cytometry |
| PE conjugated goat anti-rabbit IgG (H+L) cross adsorbed | Thermo Fisher Scientific | Cat# P-2771MP | 1:250 | Flow cytometry |
| TCF1/TCF7 rabbit polyclonal antibody | Proteintech | Cat# 14464-1-AP | 1:1,000 | Western blotting |
| TBX21/T-bet rabbit polyclonal antibody | Proteintech | Cat# 13700-1-AP | 1:1,000 | Western blotting |
| RUNX3 rabbit polyclonal antibody | Proteintech | Cat# 27099-1-AP | 1:1,000 | Western blotting |
| CD44 (E7K2Y) XP rabbit monoclonal antibody | Cell Signaling Technology | Cat# 37259 | 1:1,000 | Western blotting |
| IFN-γ (D3H2) XP rabbit monoclonal antibody | Cell Signaling Technology | Cat# 8455 | 1:1,000 | Western blotting |
| HDAC1 (D5C6U) XP rabbit monoclonal antibody | Cell Signaling Technology | Cat# 34589 | 1:1,000 | Western blotting |
| IR-Dye® 800 CW goat anti-mouse IgG | LI-COR Biosciences | Cat#926-32210 | 1:20,000 | Western blotting |
