# Supplementary material for: A viral noncoding RNA is a master regulator of gene expression that defines host cell identity and function
Source: Nucleic Acids Res. 2026 May 11;54(9):gkag472. doi: 10.1093/nar/gkag472 (PMC13158664; doi:10.1093/nar/gkag472)
Supplement: gkag472_Supplemental_Files [file gkag472_supplemental_files.zip › SuppMaterials_Gorbea et al_2025_clean.docx]

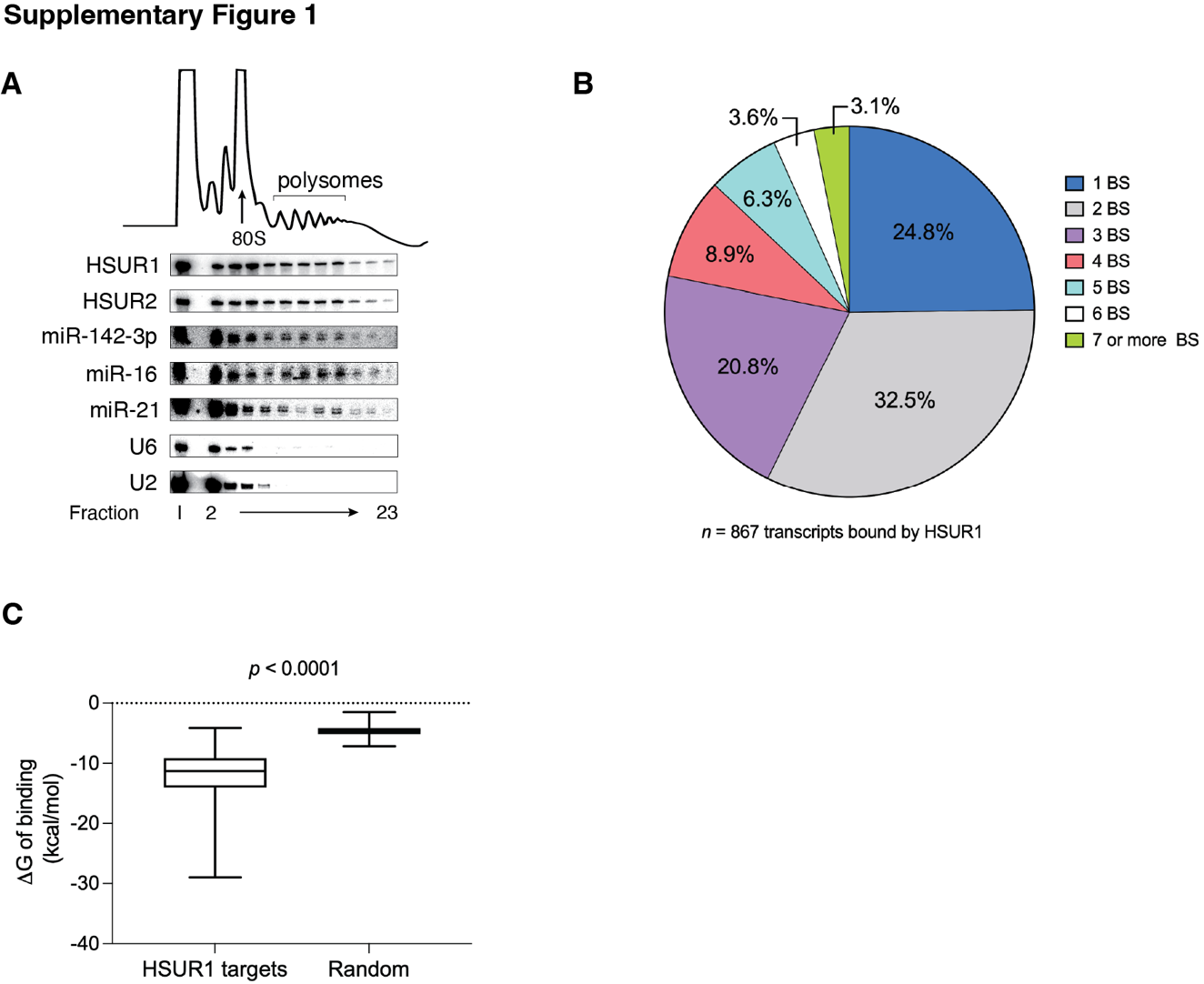


**Supplementary Figure 1. HSUR1 associates with mRNAs in HVS-transformed marmoset T cells.**

**(A)** Northern blot analysis of the indicated miRNAs and snRNAs in cytoplasmic extracts from HVS-transformed marmoset T cells (cj319-WT) fractionated by sucrose-gradient sedimentation. HSUR1 cofractionated with cytoplasmic mRNPs, consistent with its association with mRNAs. **(B)** Distribution of HSUR1 binding peaks across target mRNAs identified by iRICC, showing multiple discrete interaction sites per transcript. **(C)** Average free energy of binding (ΔG) of interactions between HSUR1 and HSUR1 binding sites (*n* = 313) compared with the mean ΔG of length-matched, random 3′UTR sequences (Control).


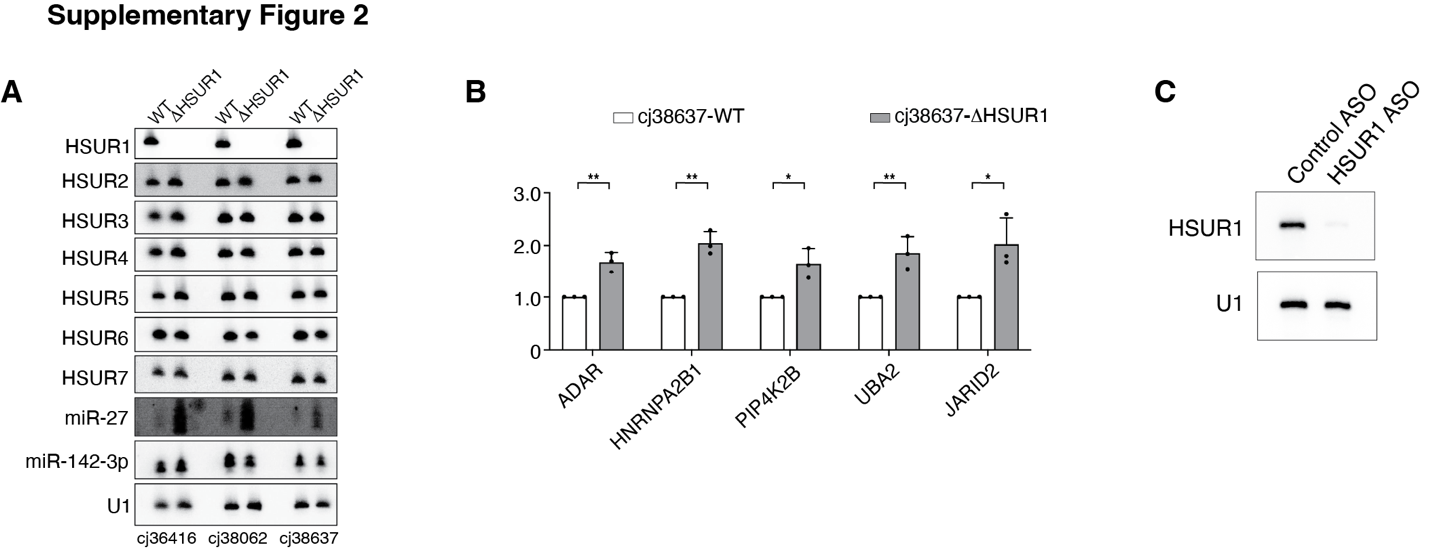


**Supplementary Figure 2. HSUR1 destabilizes target mRNAs through their 3′UTRs.**

**(A)** Northern blot analysis for miRNA and HSUR expression in total RNA isolated from cj36416, cj38062, or cj38637 cells transformed with either wild-type (WT) HVS-A11 or a mutant version lacking the HSUR1 gene (ΔHSUR1). U1 snRNA provides a loading control. **(B)** Quantification of three technical replicates of Western blots as shown in Figure 2B. (**C)** Northern blot analyses of HSUR1 and U1 (loading control) snRNAs in total RNA from cj319-WT cells transiently transfected with either control ASO or HSUR1 ASO. * *P* <0.05. ** *P* < 0.01.


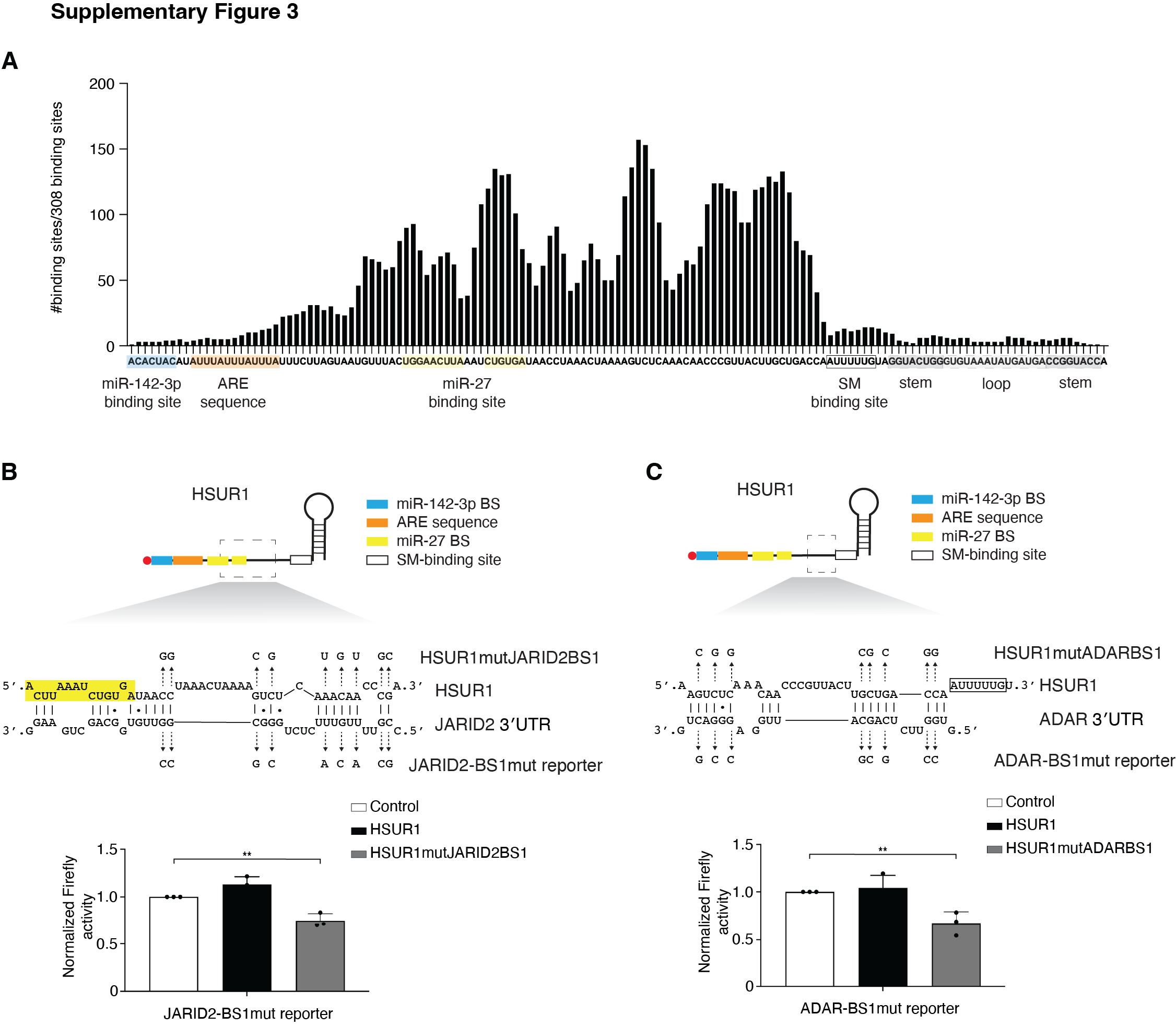


**Supplementary Figure 3. HSUR1 regulates target mRNAs through sequences defined by iRICC-seq.**

**(A)** HSUR1 exhibits flexibility in binding site recognition within 3′UTRs. The graph shows the number of times each HSUR1 nucleotide participates in base pairing with sites located in 3′UTRs of target mRNAs. Annotated features include AREs, miRNA-binding sites, and the SM-binding sequence. **(B, C)** Same as in Figure 3D for JARID2-BS1mut (B) and ADAR-BS1mut (C) reporters. ***P* < 0.01.


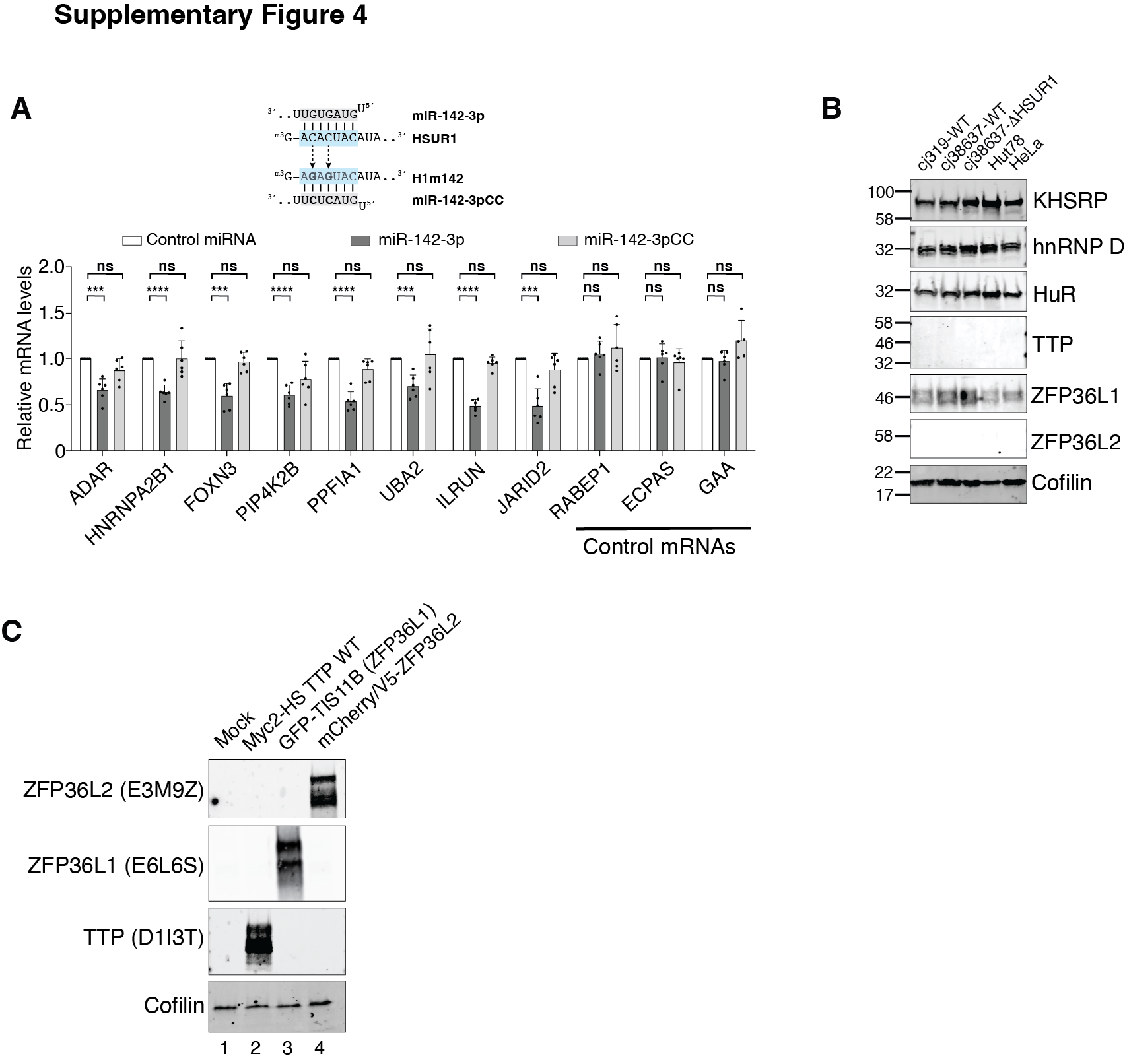


**Supplementary Figure 4. *Trans*-acting factors involved in HSUR1-mediated mRNA repression.**

**(A)** Same as Figure 4B with HeLa-H1 cells. **(B)** Western blot analyses of selected ARE-binding proteins (ARE-BPs) in the cell lines used in this study. Equal amounts of total protein were loaded; cofilin serves as a loading control. **(C)** Western blots using antibodies against TTP (D1I3T), ZFP36L1 (E6L6S), or ZFP36L2 (E3M9Z) on extracts from mock-transfected HeLa cells (lane 1) or cells transfected with plasmids expressing Myc₂-tagged TTP (lane 2), GFP-tagged ZFP36L1 (lane 3), or V5-tagged ZFP36L2. Cofilin serves as a loading control. ****P* < 0.001; *****P* < 0.0001.


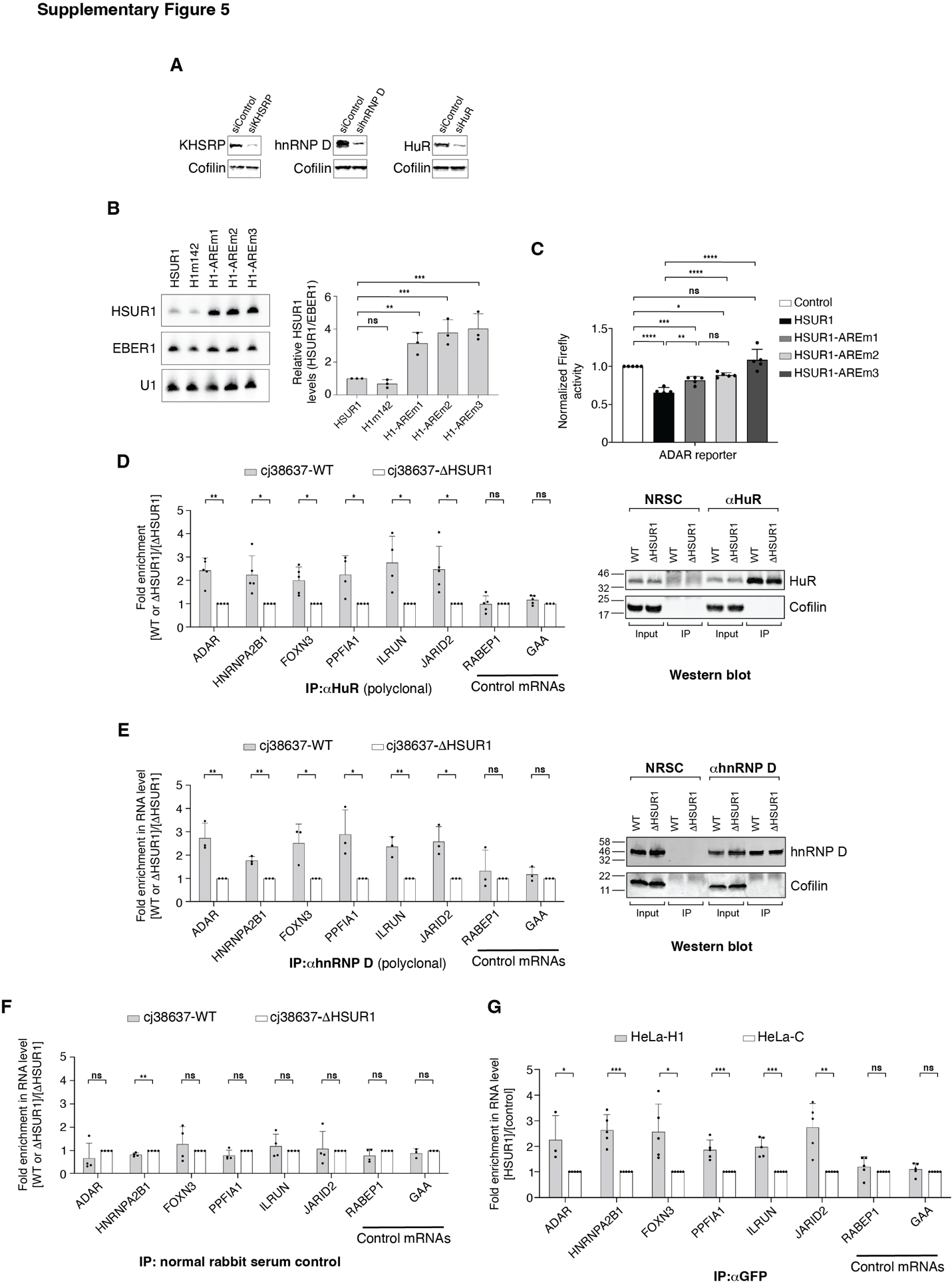


**Supplementary Figure 5. Role of HSUR1’s ARE in HSUR1 stability and HSUR1-mediated mRNA repression.**

**(A)** Western blot analyses of KHSRP, HuR, and hnRNP D in extracts from HeLa-H1 cells transiently transfected as described in Figure 5A. Cofilin serves as a loading control. **(B)** Northern blot analyses of HeLa cells transiently co-transfected with plasmids expressing wild-type HSUR1 or the indicated mutant versions of HSUR1, together with a plasmid expressing EBER1 as a transfection control. U1 snRNA serves as a loading control. Right, quantification of three independent experiments; EBER1 signal was used for normalization. **(C)** Same as in Figure 5C, using the ADAR luciferase reporter. **(D, E)** Analysis of HuR (D) and hnRNP D (E) association with HSUR1 target mRNAs, performed as in Figure 5E. **(F)** Immunoprecipitation using normal rabbit serum control. **(G)** Enrichment of HSUR1 target and control mRNAs in GFP immunoprecipitates from HeLa-C or HeLa-H1 cells transiently co-transfected with GFP-TIS11B (ZFP36L1) and miR-142-3p (see Supplementary Figure 4C). Data represent mean ± SD from three or more independent experiments. **P* < 0.05; ***P* < 0.01; ****P* < 0.001; *****P* < 0.0001.


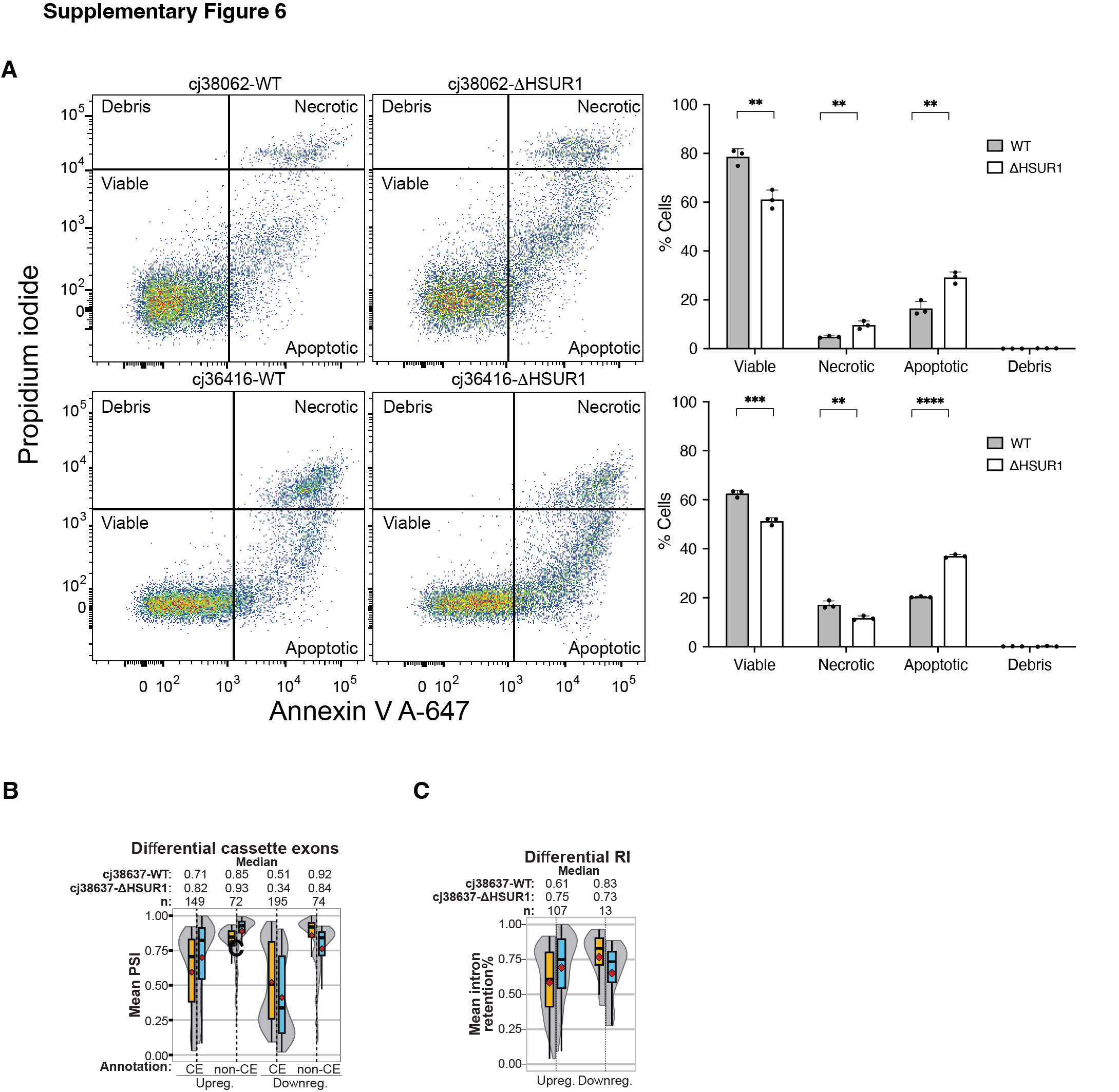


**Supplementary Figure 6. HSUR1 inhibits apoptosis and regulates splicing in HVS-transformed marmoset T cells.**

**(A)** Same as in Figure 6B for cj38062 and 36416 cells. (**B)** Distribution of percent spliced-in (PSI) values for differential cassette exons expressed in cj38637-WT and cj38637-ΔHSUR1 cells. CE: cassette exons. Non-CE: not known to be cassette exons. **(C)** Distribution of PSI values for differentially retained introns in cj38637-WT and cj38637-ΔHSUR1 cells.

** *P* < 0.01, *** *P* <0.001, **** *P* <0.0001.


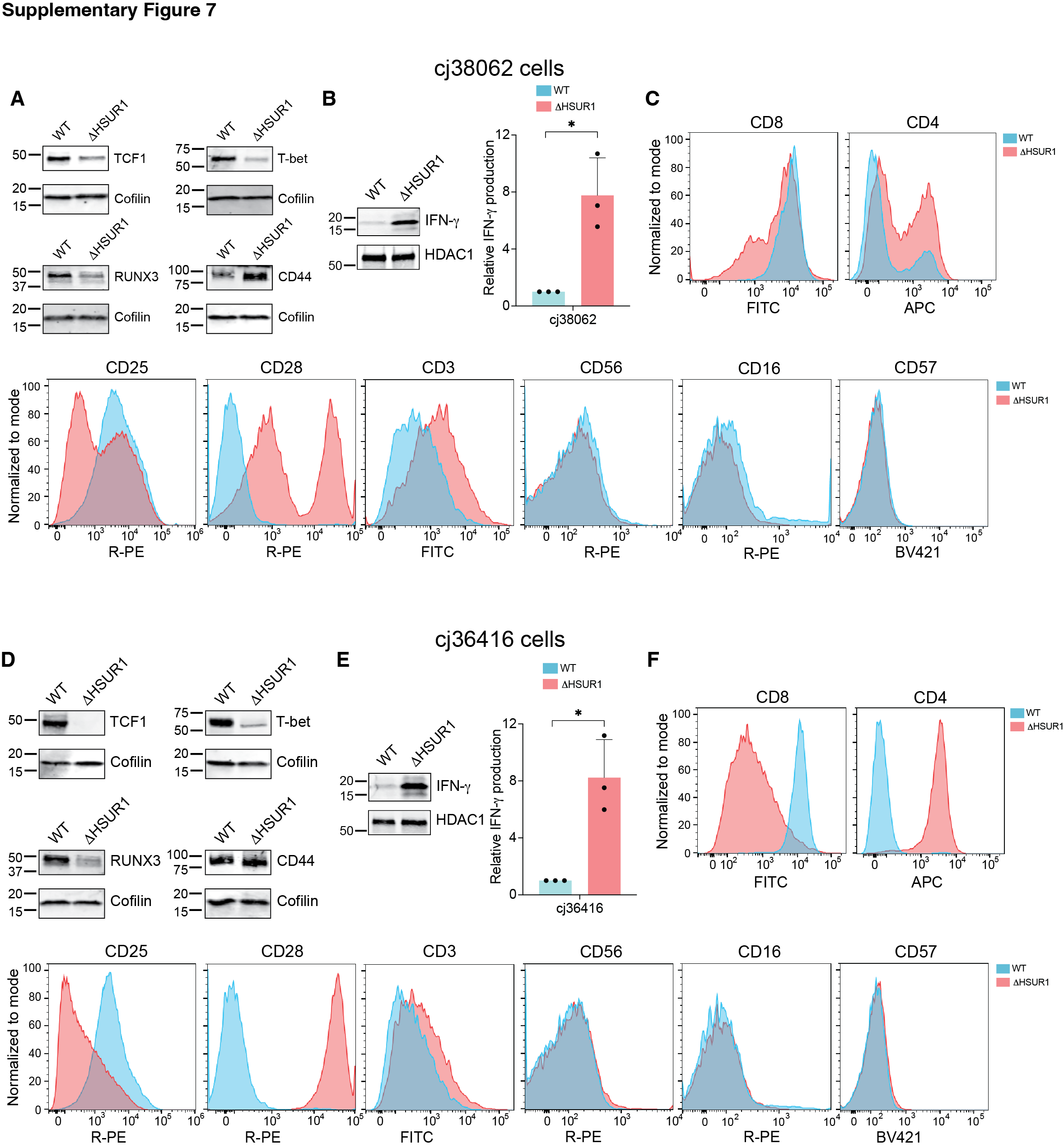


**Supplementary Figure 7. HSUR1 defines cell identity and function.**

**(A, B)** Same as in Figures 7A and 7B with cj38062 cells. **(C)** Same as in Figure 7E with cj38062 cells. **(D, E)** Same as in Figure 7A and 7B with cj36416 cells. **(F)** Same as in Figure 7E with cj36416 cells. **P* < 0.05.


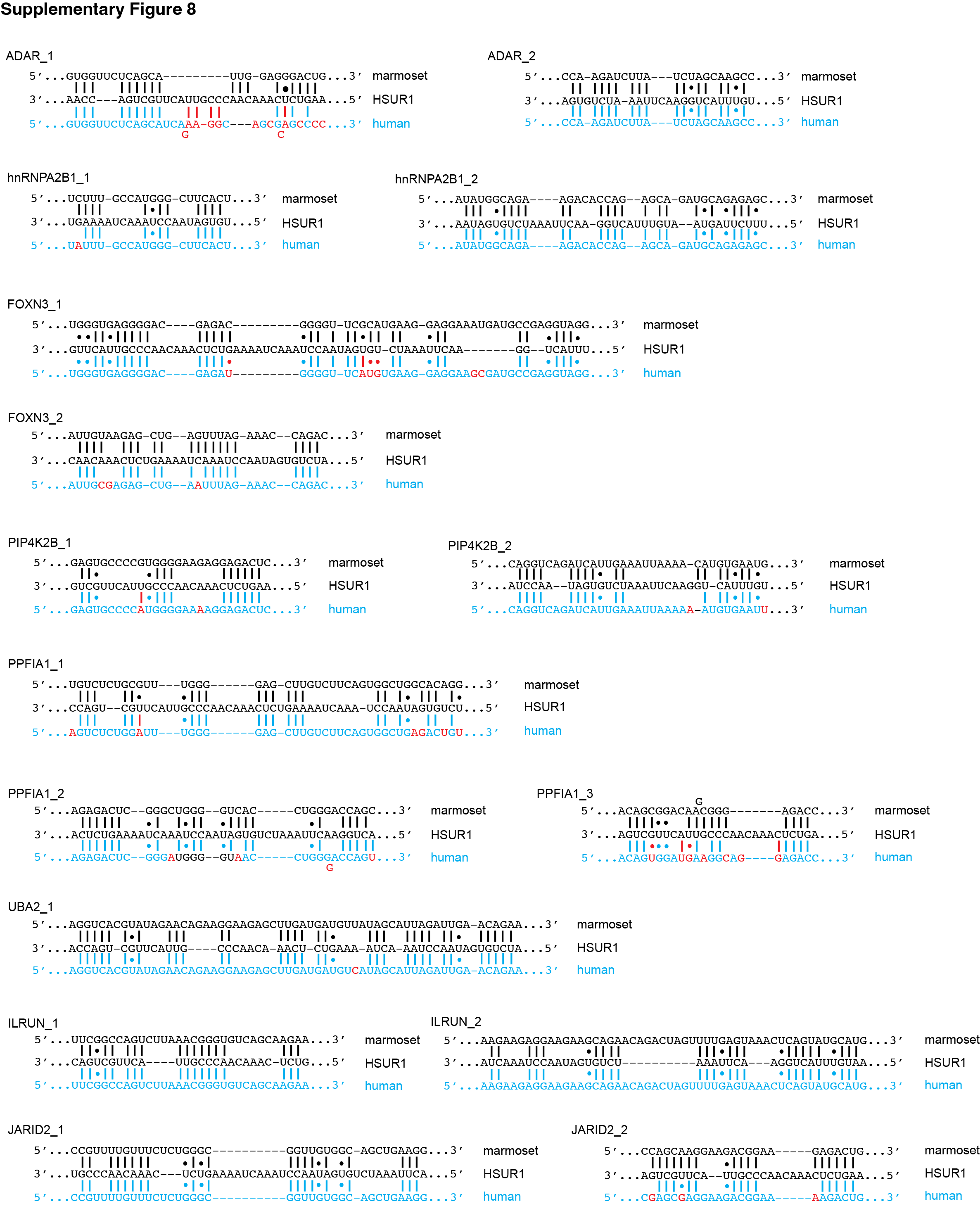


**Supplementary Figure 8. HSUR1 binding sites are conserved in human genes.**

Marmoset (black) and human (blue) sequences or HSUR1 binding sites identified by iRICC in target genes analyzed in Figure 2. Non-conserved nucleotides are shown in red in the human sequences.


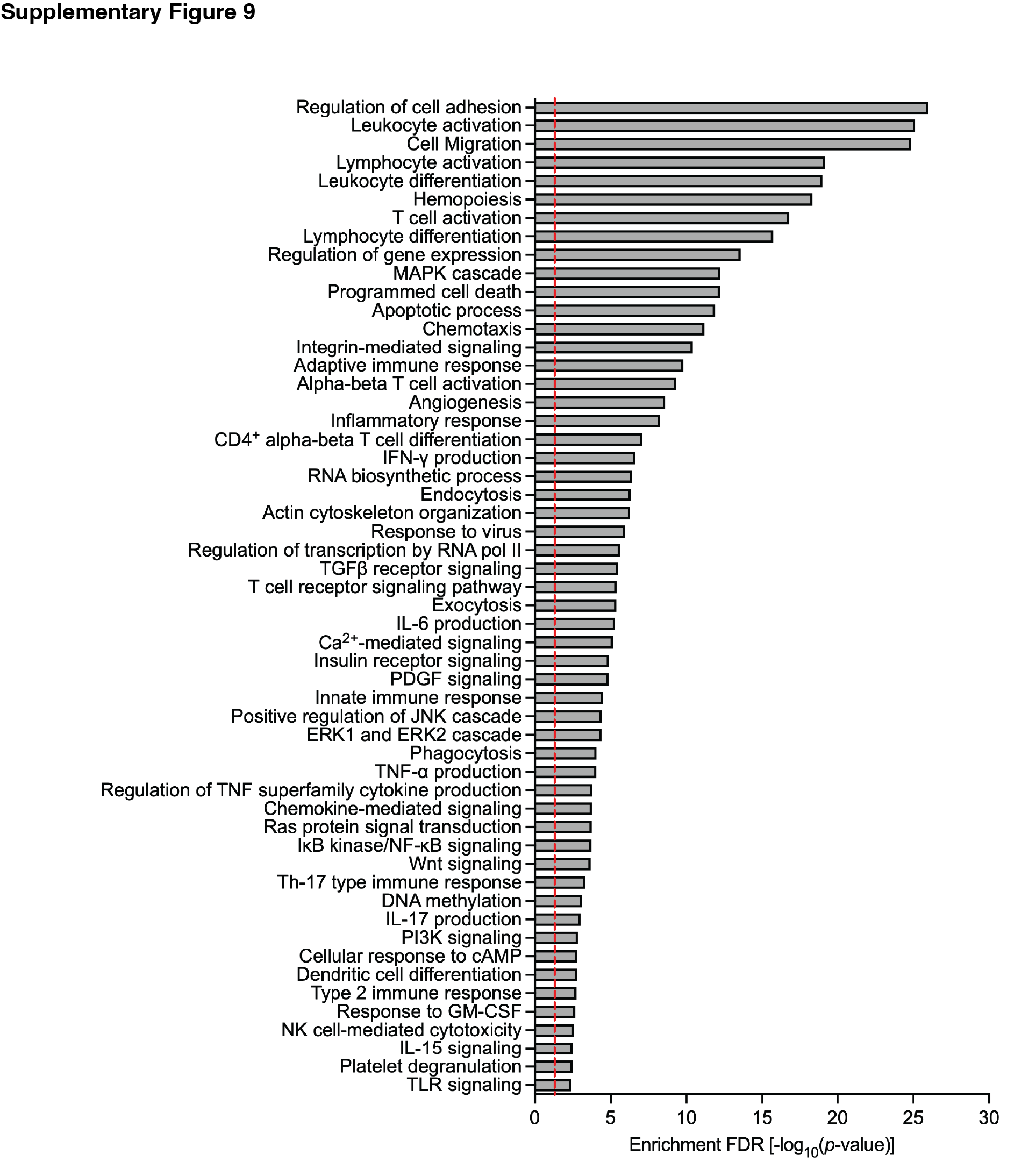


**Supplementary Figure 9. HSUR1 reprograms host gene expression.**

**(A)** Gene ontology analyses of differentially expressed genes in cj38637-WT versus cj38637-ΔHSUR1 cells. Significant enrichment is indicated by the red dashed line.


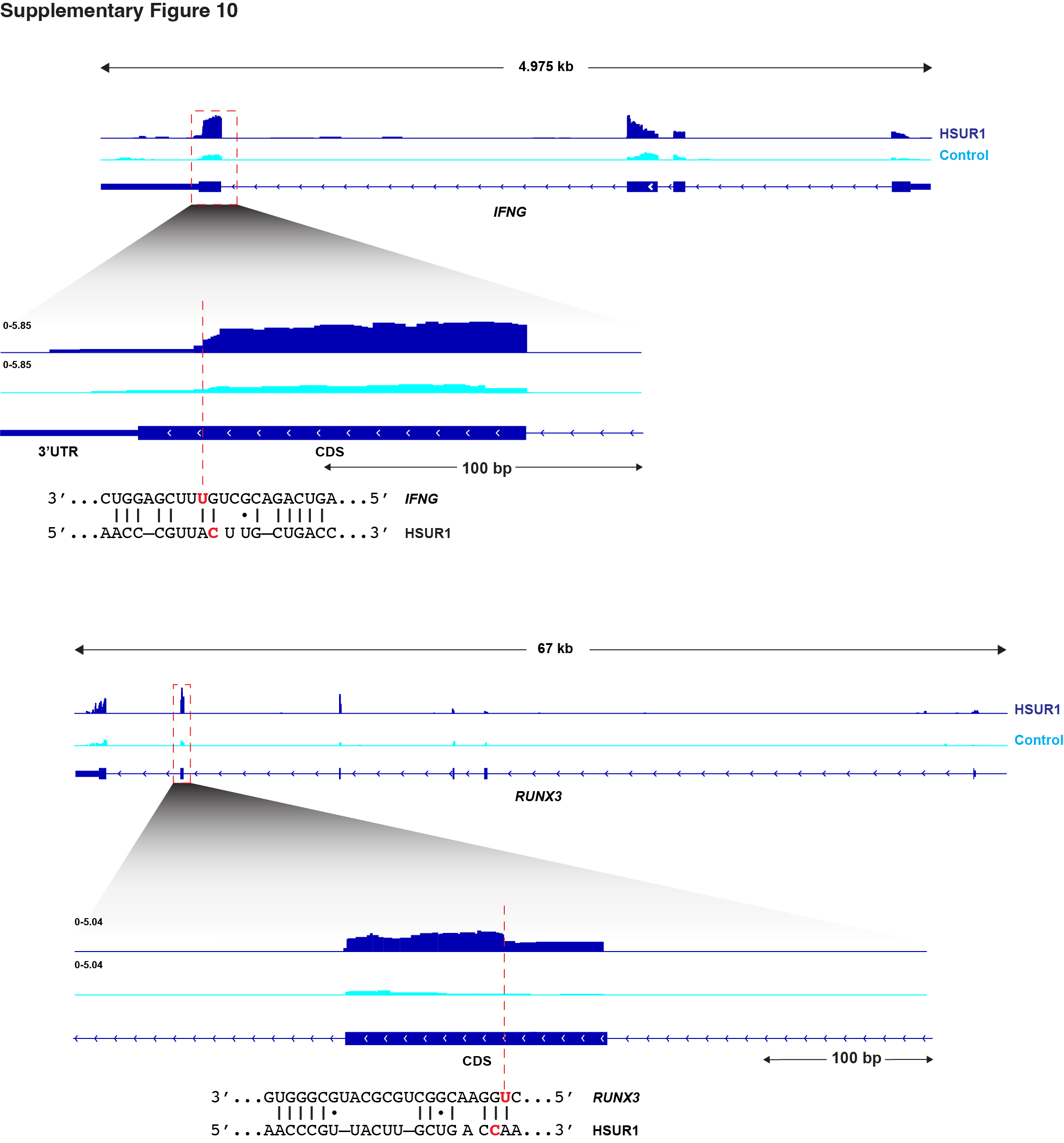


**Supplementary Figure 10. HSUR1 binding to coding sequences.**

Zoomed-in view of iRICC tracks for *IFNG* and *RUNX3* genes for HSUR1 (blue) and Control (cyan) samples. HSUR1 and Control tracks are shown at the same scale. Numbers in zoomed-in tracks indicate relative abundance normalized by unique alignment read count. Dashed red line denotes abrupt drop of aligned indicating site of crosslinking. Predicted base pairing between HSUR1 and target sequences adjacent to the site of crosslinking are shown. Putative psoralen-crosslinked nucleotides are shown in red.


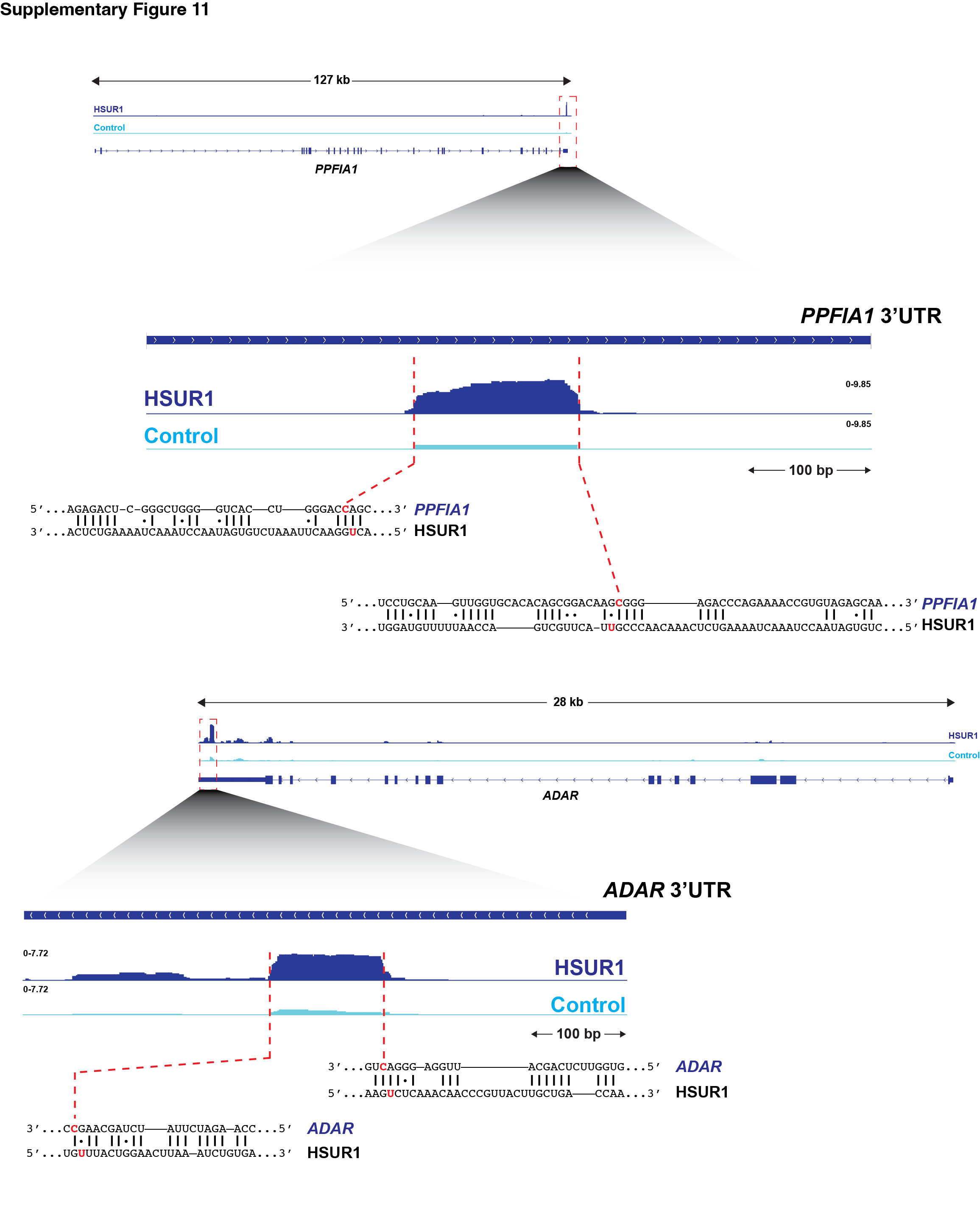


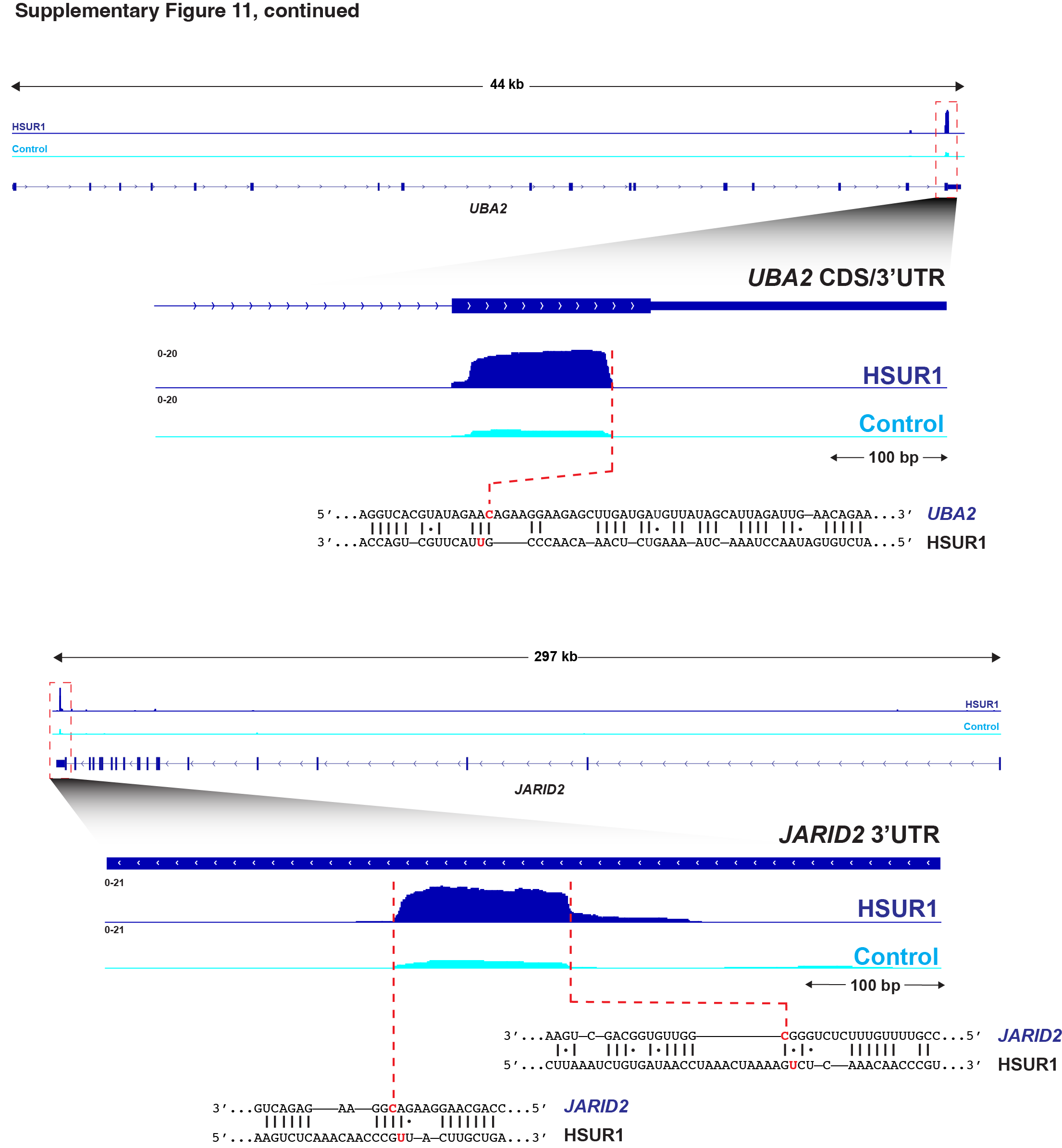


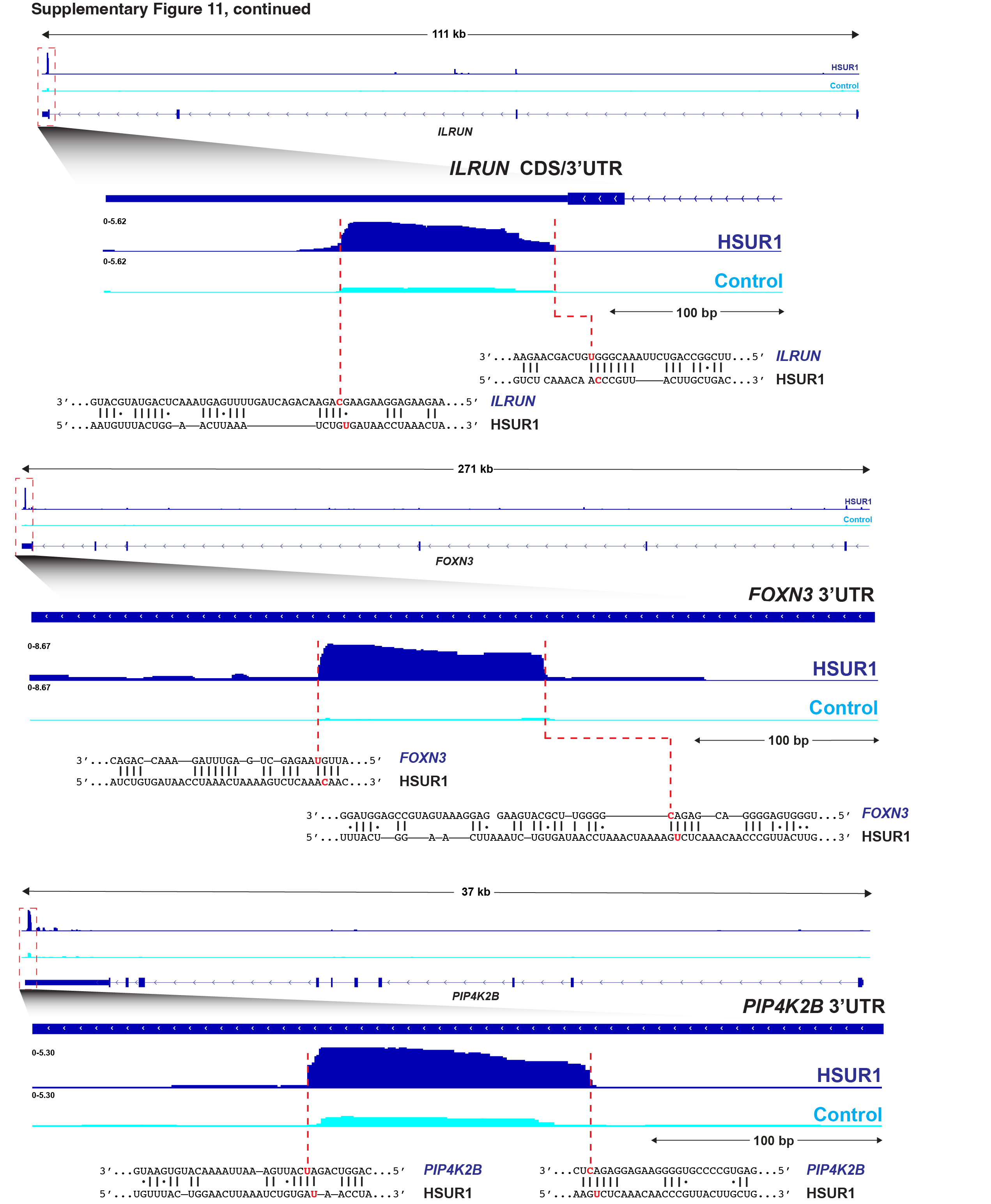


**Supplementary Figure 11. HSUR1 binding in 3′UTRs.**

Zoomed-in view of iRICC tracks for *PPFIA1, ADAR, UBA2, JARID2, ILRUN, FOXN3 and PIP4K2B*  genes for HSUR1 (blue) and Control (cyan) samples. HSUR1 and Control tracks are shown at the same scale. Numbers in zoomed-in tracks indicate relative abundance normalized by unique alignment read count. Dashed red line denotes abrupt drop of aligned indicating site of crosslinking. Predicted base pairing between HSUR1 and target sequences adjacent to the site of crosslinking are shown. Putative psoralen-crosslinked nucleotides are shown in red.
